# Targeting miR-337 mitigates disuse-induced bone loss

**DOI:** 10.1038/s41421-025-00822-z

**Published:** 2025-08-26

**Authors:** Jiao Li, Ding Ma, Chunxue Zhang, Xueling Zheng, Ruihan Hao, Bin Zuo, Fei Xiao, Yang Li, Yuhang Liu, Zhouyi Duan, Yao Xiong, Orion R. Fan, Wenmin Zhu, Liming Dai, Bingjun Zhang, Yi Eve Sun, Xiaoling Zhang

**Affiliations:** 1 Department of Orthopedic Surgery, Xinhua Hospital, Shanghai Jiao Tong University School of Medicine, Shanghai, China; 2 Department of Cell Biology, Zunyi Medical University, Zunyi, Guizhou China; 3 Shanghai Institute of Stem Cell Research and Clinical Translation, Shanghai East Hospital, School of Medicine, Tongji University, Shanghai, China; 4 Department of Orthopedics, Shanghai Sixth People’s Hospital Affiliated to Shanghai Jiao Tong University School of Medicine, Shanghai, China; 5 National Facility for Translational Medicine (Shanghai), Shanghai, China

**Keywords:** Mechanisms of disease, Mesenchymal stem cells, miRNAs

## Abstract

Disuse-induced bone loss occurs in long-term bed-ridden patients and in astronauts during spaceflight. The underlying mechanisms are poorly understood. In a rodent model of disuse-induced bone loss (called hindlimb unloading (HU)), we observed that decreased numbers of leptin receptor (LepR) positive mesenchymal stem cells (MSCs) in adult bone marrow, contribute to bone loss. MicroRNA-337-3p (miR-337) was upregulated in MSCs upon HU and inhibited MSC proliferation by directly targeting IRS-1 to suppress the PI3kinase-Akt-mTOR pathway. Piezo1 was the upstream receptor for sensing mechanical stress and regulated miR-337 through the Hippo-YAP signaling pathway. Remarkably, the knockout of miR-337 significantly attenuated HU-induced, but not ovariectomy-induced, bone loss by increasing MSC proliferation and osteogenesis. Finally, the transplantation of miR-337^-/-^ MSCs into wild-type HU mice was sufficient to mitigate bone loss. These findings reveal the cellular and molecular mechanisms underlying disuse-induced bone loss and highlight a feasible therapeutic strategy to prevent disuse- or microgravity-induced bone loss on Earth and during spaceflight.

## Introduction

Disuse-induced bone loss occurs on Earth in long-term bed-ridden patients and in astronauts during long-term spaceflight owing to microgravity^1–5^. Under these conditions reduced mechanical stress can cause significant bone loss, which subsequently leads to additional systemic disturbances in the human body^6–8^. Bone is a dynamic tissue that undergoes constant modeling and remodeling in response to mechanical loading^9–12^. Osteocytes were the first identified primary mechanosensors in bone, regulating the bone remodeling process by producing soluble factors such as bone morphogenetic proteins (BMPs), sclerostin, receptor activator of nuclear factor κB ligand (RANKL), and osteoprotegerin (OPG) in response to changes in mechanical loading^13–18^. In recent years, additional bone cell types, as well as leptin receptor (LepR) positive mesenchymal stem cells (MSCs)^19^, have been shown to be capable of mechanical sensing^20–24^. Moreover, mechanical unloading-induced bone loss may also be attributed to changes in MSC function^25^. MSCs represent one of the major sources for the initiation of osteogenic processes. The underlying molecular mechanisms by which mechanical unloading decreases the number of MSCs and whether prevention of MSC loss may attenuate disuse osteoporosis remain to be determined.

Emerging evidence highlights the critical role of microRNAs in skeletal muscle systems. These small noncoding RNAs regulate bone homeostasis by fine-tuning osteogenic differentiation^26,27^, osteoclast activity^28–30^, and MSC fate determination^31–33^. Notably, mechanical loading dynamically modulates miRNA expression profiles in various cells. For example, fluid shear stress downregulates miR-103a to promote osteoblast mineralization^34^, while compressive loading induces miR-33-5p expression to enhance osteoblast differentiation^35^. Our previous work first identified MicroRNA-337-3p (miR-337) as a mechanical stress-sensitive regulator in tendon stem cells, where it mediates osteogenic differentiation through the direct targeting of IRS-1 under cyclic tensile stress^36^. This discovery identified miR-337 as a potential key mediator that translates mechanical signals into cellular responses across musculoskeletal tissues.

Recent studies have established Piezo1 as a pivotal mediator of cellular mechanical stress sensing and signaling, with its ion channel activity directly linking extracellular forces to intracellular biochemical responses, and regulating osteogenesis^25,37^, angiogenesis^38,39^, and stem cell differentiation^40,41^. While Piezo1 activation has been shown to promote osteoblast differentiation through YAP/TAZ signaling^42^, its specific function in maintaining MSC proliferation under physiological loading remains unspecified. Our study reveals a novel regulatory axis in which Piezo1 governs MSC proliferation through miRNA regulation, establishing previously unrecognized crosstalk between ion channel signaling and post-transcriptional control in mechanical stress transduction.

Current strategies to combat disuse osteoporosis primarily focus on suppressing bone resorption or enhancing mechanical loading^43,44^. However, these approaches do not comprehensively address the fundamental issue of MSC depletion in the pathological bone marrow (BM) microenvironment. The development of targeted interventions to preserve MSC populations under mechanical unloading conditions represents an urgent unmet clinical need, particularly for spaceflight applications where conventional weight-bearing countermeasures are impractical. Here we propose a novel mechanical regulatory axis centered on miR-337 that couples upstream mechanical stress sensing by the Piezo1/YAP/TEAD pathway with the downstream control of MSC proliferation through IRS-1/PI3K/Akt/mTOR signaling. The genetic ablation of miR-337 specifically rescued hindlimb unloading (HU)-induced bone loss by restoring MSC proliferation and osteogenic differentiation. Additionally, the transplantation of miR-337-deficient MSCs into wild-type mice subjected to HU robustly attenuated disuse-induced bone deterioration. Based on these findings, our data establish a foundation for the targeted inhibition of miR-337 as a novel therapeutic approach to preserve MSC populations and osteogenic potential under mechanical disuse conditions.

## Results

### The number of MSCs is markedly decreased in the BM of a rat model of HU

**Fig. 1:**
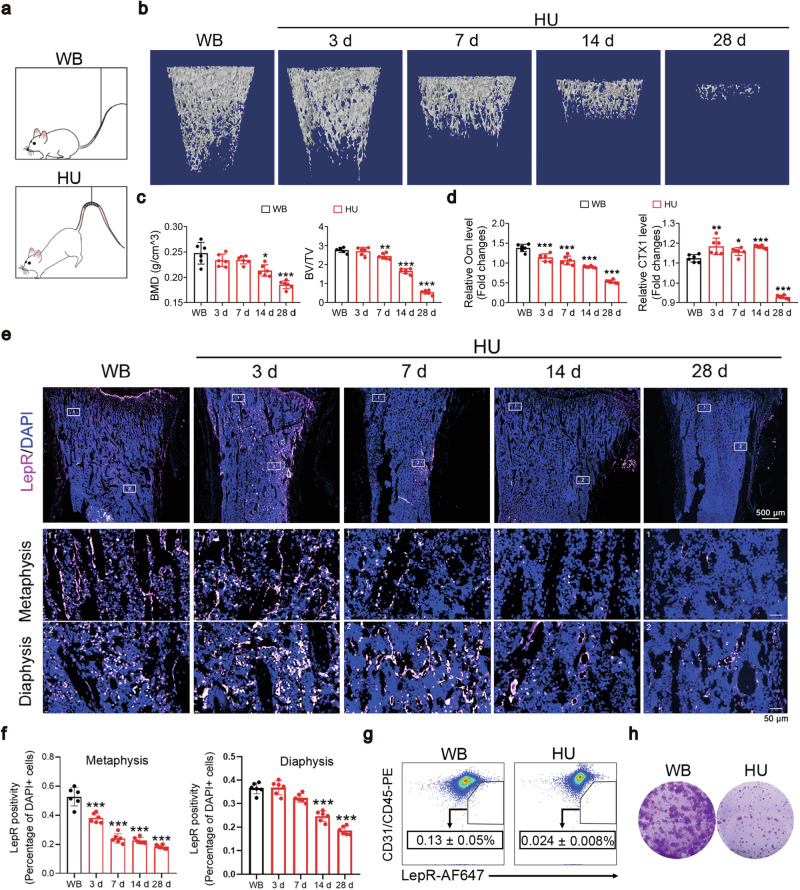
The MSC frequency was markedly reduced in BM of HU rats. **a** Schematic of HU models and WB controls. Representative micro-CT images (**b**) and quantification of three-dimensional microstructural parameters (**c**) of femurs from HU rats and WB controls. **d** Relative serum Ocn and CTX1 levels in WB and HU rats. The data are presented as the fold changes relative to those of the WB samples. **e** Representative images of tibia sections from rats subjected to HU for 7 days and stained with LepR. **f** Quantification of LepR^+^ cells. The data are presented as percentages of positively stained cells to total cells (DAPI-stained nuclei) *P < 0.05, **P < 0.01, and ***P < 0.001 compared with WB (one-way ANOVA with Dunnett’s post hoc test), *n* = 6 per group. The data are presented as the means ± SEM. **g** Flow cytometry analysis showing the percentage of CD45/CD31^–^LepR^+^ cells in the BM of WB rats and rats suspended for 7 days. The data are presented as the means ± SD, *n* = 6 per group, and are represe*n*tative of three independent experiments. **h** Representative image of CFU clones formed by the BM cells derived from rats subjected to unloading for 7 days. The number of clones larger than 40 cells was calculated.

The HU rodent model has been extensively used to study various physiological responses to certain aspects of spaceflight as well as long-term consequences of being bedridden, including bone loss^2^. We used an HU rat model established via tail suspension (Fig. 1a). Micro-CT imaging revealed marked bone loss (Fig. 1b, c) by day 14 after unloading. Alkaline phosphatase (ALP) staining and serum osteocalcin (Ocn) levels indicated that the bone remodeling balance shifted to favor bone resorption over bone formation as early as day 3 (Fig. 1d and Supplementary Fig. S1a, b). Bone formation activity was barely detectable seven days after unloading, whereas bone resorption activity remained high until day 14 (Fig. 1d and Supplementary Fig. S1a, b).

As LepR^+^ MSCs play critical roles in maintaining the balance between bone formation and resorption^19,45^, we quantified the numbers of LepR^+^ cells in bone sections from weight-bearing (WB) and HU rats by immunofluorescence labeling. Three days after HU, the number of LepR^+^ cells in the metaphysis (the part of long bones that contains the growth plate) was dramatically decreased, whereas changes in the diaphysis (the midsection of long bones that contains BM became significant only from day 14 onward (Fig. 1e, f).

Since LepR^+^ cells are heterogeneous^19,45,46^, we performed a flow cytometry analysis to study LepR^+^ MSCs in BM, which are CD45 and CD31 double negative, and found that the percentage of MSCs (CD45^−^CD31^−^LepR^+^) decreased by more than 50% (HU vs. WB) upon unloading (Fig. 1g). Consistently, the colony-forming unit (CFU) frequency of the MSCs also dramatically decreased by day 7 (Fig. 1h). Taken together, these results provide evidence that HU causes the loss of MSCs, which might contribute to impaired new bone formation under mechanical unloading.

**Fig. 2:**
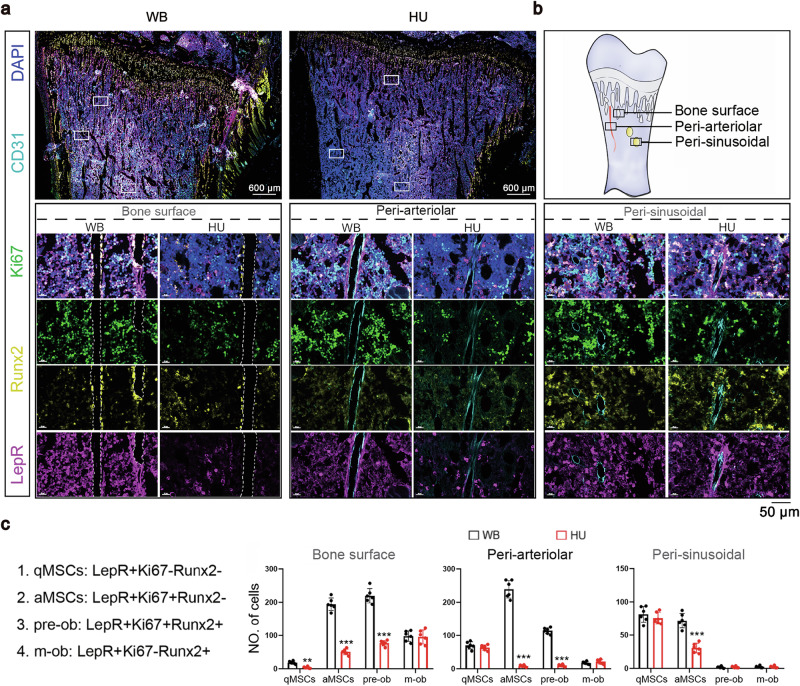
The number of proliferating LepR^+^ cells was decreased in the BM of unloaded rats. **a** Representative images of mIHC of tibia sections from WB controls and rats suspended for 7 days. Scale bars, (upper panel) 600 μm; (lower panel) 50 μm. **b** Schematic of the LepR^+^ cell niches. **c** Quantification of the 4 subsets of LepR^+^ cells in the 3 niches (< 50 μm), *n* = 6 per group.

We performed multiple immunohistochemistry (mIHC) assays to further examine the spatial distribution of MSC subsets. We used three markers (LepR, Ki67, and Runx2) simultaneously to identify MSCs in four different functional states (Fig. 2a), i.e., quiescent MSCs (qMSCs, LepR^+^Ki67^−^Runx2^−^), activated MSCs (aMSCs, lepR^+^Ki67^+^Runx2^−^), preosteoblasts (pre-obs, LepR^+^Ki67^+^Runx2^+^), and mature osteoblasts (m-obs, LepR^+^Ki67^−^Runx2^+^). We used CD31 to label arterioles (CD31^high^) and sinusoids (CD31^low^) in the BM^47^. The frequencies of qMSCs and m-obs did not significantly change in three BM regions (bone surface, arterioles, and sinusoids) (Fig. 2b) of HU rats, whereas, the frequencies of aMSCs and pre-obs were markedly decreased, with those in the peri-arteriolar regions showing the most dramatic changes (Fig. 2c). Since both aMSCs and pre-obs were Ki67^+^ dividing cells, this observation suggests that mechanical unloading specifically affects proliferating LepR^+^ MSCs.

### Mechanical stretching activates quiescent MSCs through the miR-337-PI3K-Akt axis

**Fig. 3:**
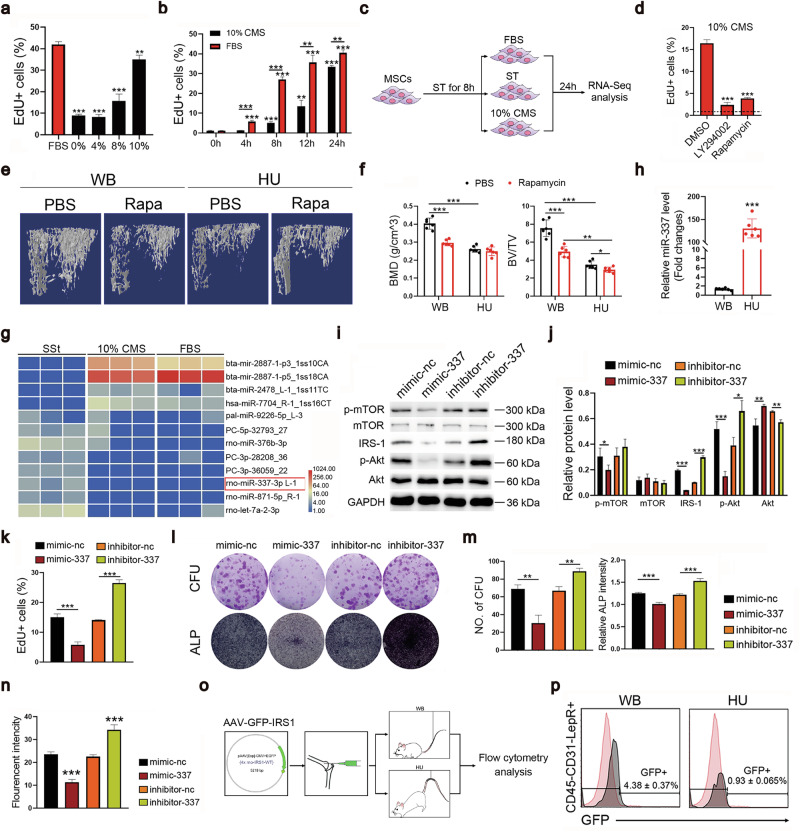
CMS-activated quiescent MSCs by the miR-337-PI3K-Akt-mTOR pathway. Flow cytometry analysis showing the percentage of EdU^+^ MSCs treated with the indicated CMS elongations (**a**) or 10% CMS (**b**) for the indicated time after serum-starvation for 8 h. **c** Schematic of the different MSC treatments groups used for the RNA-seq analysis. **d** Flow cytometry analysis of the percentage of EdU^+^ MSCs starved for 12 h and then stimulated with 10% CMS for another 24 h and treated with DMSO, LY294002, or rapamycin. Antagonistic agents were added at the time of the CMS treatment. The baseline (black dotted line) indicates the percentage of EdU^+^ MSCs before CMS treatment. **e** Rapamycin administration decreased the bone volume of WB rats as indicated by the micro-CT analysis of tibia sections from rats treated for 28 days. **f** Quantification of three-dimensional microstructural parameters from micro-CT images. **g** Heatmap of differentially expressed miRNAs that specifically target the PI3K-Akt pathway in MSCs treated with 10% CMS or FBS for 24 h after 8 h of serum starvation. **h** Quantitative RT-PCR was used to measure the expression of miR-337 in de novo isolated LepR^+^ cells from rats suspended for 7 days as well as WB controls. **i** Representative Western blot images showing the activation of the PI3K-Akt-mTOR pathways. The cells were transfected with the miR-337 mimic or inhibitor before they were subjected to 10% CMS-stimulated proliferation. **j** Quantification of the relative protein levels was normalized to the intensity of GAPDH in 3 independent experiments. **k** Flow cytometry analysis showing the percentage of EdU^+^ cells after being transfection with the miR-337 mimic or inhibitor, followed by 10% CMS stimulation for 24 h. **l** Representative images of CFU-F and ALP staining. MSCs were transfected with the miR-337 mimic or inhibitor as indicated for 24 h before the CFU-F and osteogenic assays were performed. **m** Quantification of CFU and ALP staining intensities shown in (**l**). **n** Flow cytometry analysis revealed that the fluorescent intensity of the GFP-IRS1 reporter was regulated by miR-337. **o** Schematic of the in vivo reporter assay. **p** Flow cytometry analysis showing the fluorescence intensity of GFP in CD45^–^/CD31^–^/LepR^+^ cells in rats suspended for 7 days after an intramedullary injection of the AAV-GFP-IRS1 reporter. Statistical significance was assessed by Student’s *t*-test of the results from 3 independent experiments. *n* = 3 per group. *P < 0.05, **P < 0.01, ***P < 0.001. The data are presented as means ± SD. The miR-337 expression levels were normalized to those of U6.

We utilized in vitro cultures of BM-derived MSCs, 99.5% of which were LepR^+^, to define the molecular mechanisms by which HU impairs the proliferation of LepR^+^ MSCs (Supplementary Fig. S1c). The proliferation of these MSCs is serum-dependent (Supplementary Fig. S1d, e). Upon serum starvation (SSt), cultured MSCs became quiescent and proliferated very slowly, however, this quiescent state could be rapidly converted into an activated state upon serum replacement (Supplementary Fig. S1d, e). Since the arteriolar wall reportedly transmits mechanical signals to surrounding LepR^+^ MSCs, we explored whether cyclic mechanical stretching (CMS) could also activate qMSCs in culture after SSt^25^. Using an FX-5000T Flexercell Tension Plus unit on in vitro cultured MSCs, which had undergone SSt for eight hours, we found that qMSCs were activated to proliferate robustly with a 10% elongation force (Fig. 3a), and were sustained for 24 h (Fig. 3b).

Transcriptomic analyses of serum (FBS) and CMS-activated MSCs (Fig. 3c) revealed differentially expressed genes (DEGs) between the CMS-stimulated and SSt conditions and between the serum-treated and SSt groups (Supplementary Fig. S2a). We detected substantially fewer DEGs between the CMS and FBS groups (Supplementary Fig. S2a), indicating that the CMS and FBS groups were more similar to each other and that both were distinct from the SSt group. More strikingly, Gene Ontology and pathway enrichment analyses of DEGs revealed that CMS and FBS stimulation activated many similar biological processes (Supplementary Fig. S2b, c), including the PI3K-Akt-mTOR pathway. This pathway has been reported to play important roles in the activation of many different types of quiescent stem cells, including hematopoietic stem cells (HSCs)^48–50^, epidermal stem cells^51^, neural stem cells^52^, and muscle stem cells^53^. Western blot analyses further confirmed the activation of the downstream mTOR protein by CMS (Supplementary Fig. S1f). Inhibition of this pathway either by the PI3K antagonist LY294002 or by the mTOR inhibitor rapamycin abrogated CMS-stimulated proliferation (Fig. 3d), confirming the pivotal role of the PI3K-Akt pathway in CMS-induced MSC activation.

We performed Western blot analyses to determine whether this pathway is also involved in regulating MSC proliferation in vivo, as well as in mediating the HU-induced depletion of MSCs, and confirmed that HU inhibited of the PI3K-Akt-mTOR pathway in BM LepR^+^ cells on Day 14 after tail suspension compared with normal WB controls (Supplementary Fig. S2d). Furthermore, the injection of rapamycin, an mTOR inhibitor, was sufficient to induce bone loss even in WB animals, similar to HU (Fig. 3e, f). Rapamycin and HU were almost equally effective, suggesting that HU-induced bone loss is likely mediated predominantly (if not exclusively) by the inhibition of the PI3K-Akt-mTOR pathway (Fig. 3e, f).

Since microRNAs can be regulated by various biological stimuli and coordinately regulate important signaling pathways, we analyzed our transcriptomic data for altered microRNA expression in MSCs subjected to SSt, CMS, or FBS treatment. Among the top differentially expressed miRNAs that are known to target the PI3K-Akt-mTOR pathway (Fig. 3g), miR-337 was highly expressed. In vivo, HU caused a more than 100-fold increase in miR-337 expression in BM LepR^+^ cells on day 7 after tail suspension compared with that in WB controls (Fig. 3h). We previously reported that miR-337 is mechanically sensitive and can mediate the effects of CMS on tendon stem cell differentiation by directly binding to the 3’ UTR of the insulin receptor substrate 1 (IRS-1) mRNA to inhibit its protein translation, which in turn, suppresses the PI3K-Akt-mTOR pathway^36,54^. Using both gain- (mimic-337) and loss-of-function (inhibitor-337) assays, we further confirmed that this microRNA was capable of inhibiting the PI3K-Akt-mTOR pathway as well as MSC proliferation and osteogenesis (Fig. 3i–m). Interestingly, in cultured MSCs, miR-337 expression was inhibited by FBS, CMS, and several growth factors, but not by BMP2 or TGF-β (Supplementary Fig. S2e).

We utilized an AAV vector carrying a GFP reporter with an IRS-1 3’UTR sequence potentially targeted by miR-337 to test if miR-337 also targeted the 3’ UTR of the IRS-1 mRNA in the knee joint in vivo, (Fig. 3n). The reporter AAVs were introduced into the medulla of HU or WB rats (Fig. 3o). GFP expression from this reporter was significantly inhibited in LepR^+^ MSCs of HU rats compared with those of WB rats (Fig. 3p). Thus, HU induces the expression of miR-337, which inhibits IRS-1 expression both in vitro and in vivo.

**Fig. 4:**
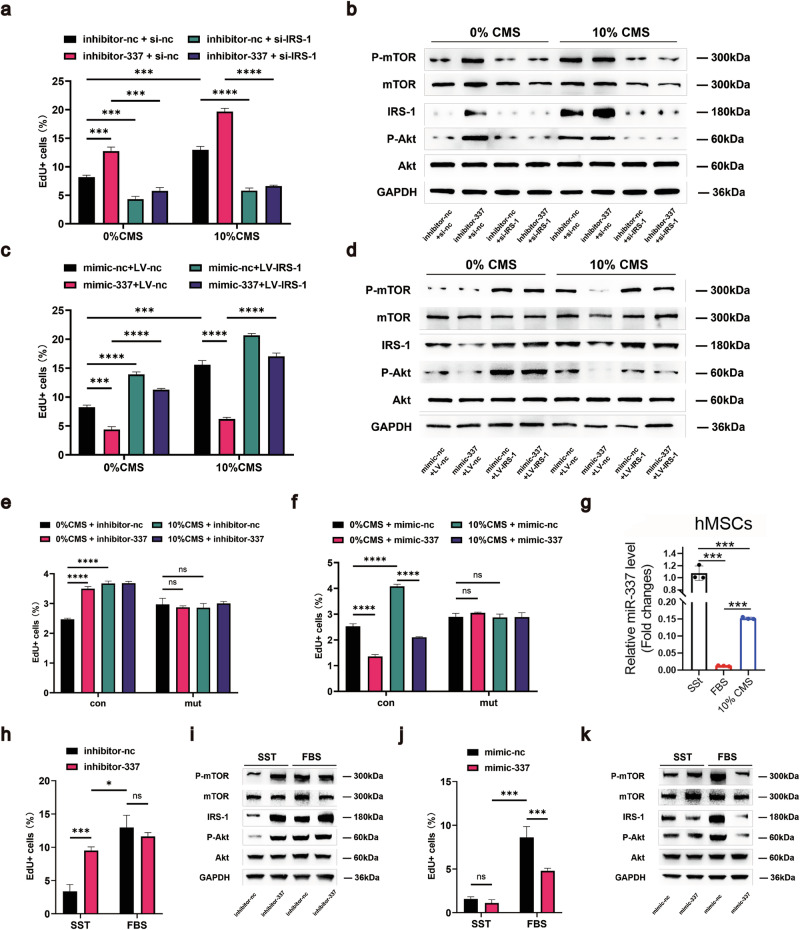
miR-337 regulates the PI3K-Akt signaling pathway through IRS-1. **a** Flow cytometry analysis showing the percentage of EdU^+^ rMSCs after transfection with the miR-337 inhibitor or IRS-1 si-RNA as indicated, followed by treatment with 0% CMS or 10% CMS for 24 h after 8 h of serum starvation. **b** Representative Western blot images showing the activation of the PI3K-Akt-mTOR pathway. rMSCs were transfected with the miR-337 inhibitor or IRS-1 si-RNA as indicated before they were subjected to 0% CMS or 10% CMS for 24 h after 8 h of serum starvation. **c** Flow cytometry analysis showing the percentage of EdU^+^ rMSCs after infection with LV-nc or LV-IRS-1 and subsequent transfection with the miR-337 mimic as indicated, followed by 10% CMS stimulation for 24 h after 8 h of serum starvation. **d** Representative Western blot image showing the activation of the PI3K-Akt-mTOR pathway. rMSCs were infected with LV-nc or LV-IRS-1 and transfected with the miR-337 mimic as indicated sequentially, followed by 10% CMS stimulation for 24 h after 8 h of serum starvation. **e**, **f** Flow cytometry analysis showing the percentage of EdU^+^ con-rMSCs or mut-rMSCs after transfection with the miR-337 inhibitor or mimic as indicated, followed by treatment with 0% CMS or 10% CMS for 24 h after 8 h of serum starvation. **g** Quantitative RT-PCR was used to measure the expression of miR-337 in hMSCs treated as indicated for 24 h after they were serum-starved for 8 h. **h**, **j** Flow cytometry analysis showing the percentage of EdU^+^ hMSCs after transfection with the miR-337 inhibitor or mimic as indicated, followed by treatment as indicated for 24 h after being serum-starved for 8 h. **i**, **k** Representative Western blot images showing the activation of the PI3K-Akt-mTOR pathway in hMSCs. The cells were transfected with the miR-337 inhibitor or mimic before being treated as indicated for 24 h after being serum-starved for 8 h. Statistical significance was assessed by Student’s *t*-test of the results from 3 independent experiments. *n* = 3 per group. *P < 0.05, **P < 0.01, and ***P < 0.001. The data are means ± SD. The miR-337 expression levels were normalized to those of U6.

We studied the effects of IRS-1 overexpression and knockdown by performing miR-337 gain- and loss-of-function assays to further elucidate the epistatic relationship between miR-337 and IRS-1-PI3K/Akt signaling pathway in rat BM LepR^+^ mesenchymal stem cells. When co-transfected with si-IRS-1, the proliferation-promoting effects of “inhibitor-337” on 0% CMS-treated MSCs were dampened, and PI3K-Akt signaling activity was also reduced (Fig. 4a, b). IRS-1 overexpression, on the other hand, counteracted “mimics-337” to rescue the proliferative ability of MSCs, while enhancing the activation of the PI3K-Akt signaling pathway (Fig. 4c, d). Additionally, we used CRISPR-CAS9 to mutate the miR-337 binding site in the IRS-1 3’ UTR, constructed IRS-1-mutant MSCs (mut-MSCs) and compared them with wild-type IRS-1-control MSCs (con-MSCs) (Supplementary Fig. S2f). Through EdU^+^ flow assays and Western blot, we observed that the proliferation and expression levels of PI3K-Akt pathway components in mutant MSCs were no longer affected by either 10% CMS or the overexpression of miR-337, unlike those of con-MSCs (Fig. 4e, f and Supplementary Fig. S2g, h). Similarly, the expression of miR-337 was affected in human MSCs (Fig. 4g) by SSt, FBS, and 10% CMS, suggesting that this regulatory pattern is conserved in human cells. Likewise, the overexpression and knockdown of miR-337 in human MSCs also affected the proliferative ability and expression levels of PI3K-Akt signaling pathway components (Fig. 4h–k and Supplementary Fig. S2i). In conclusion, mechanical stretching regulates the PI3K-Akt signaling pathway by suppressing miR-337 expression to promote the proliferation of quiescent MSCs.

### MSCs sense stress stimulation through the Piezo1-Hippo-YAP-miR-337-PI3K-Akt axis

**Fig. 5:**
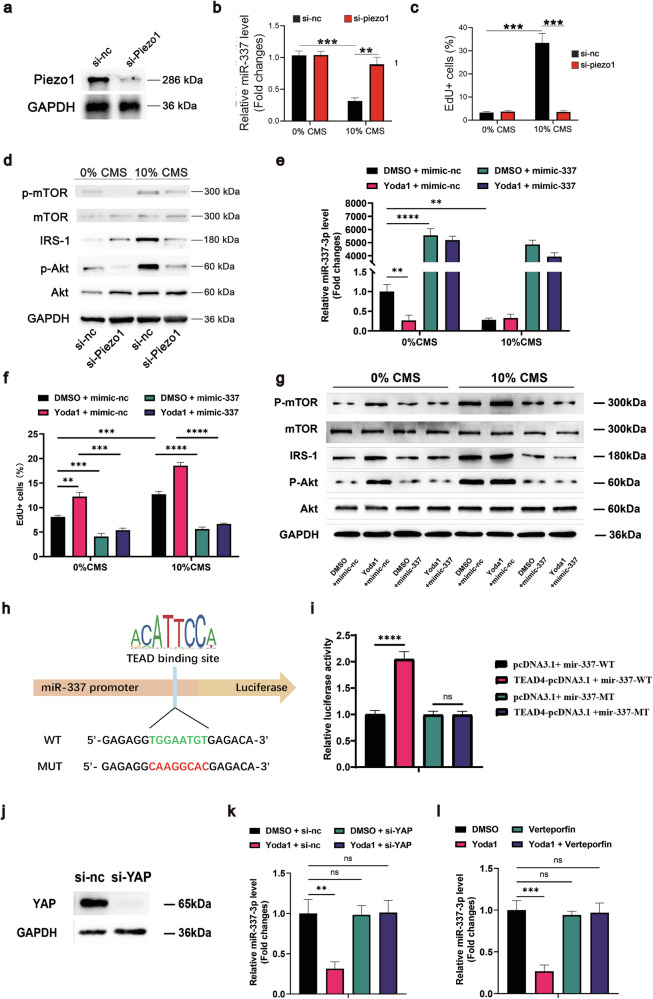
MSCs sense stress stimulation through the Piezo1-miR-337-PI3K-Akt axis. **a** Representative Western blot images showing the Piezo1 protein level at 72 h after siRNA transfection. **b** Quantitative RT-PCR analysis of the expression of miR-337 in rMSCs transfected with the Piezo1 si-RNA followed by treatment with 0% CMS or 10% CMS for 24 h after 8 h of serum starvation. **c** Flow cytometry analysis showing the percentage of EdU^+^ rMSCs after transfection with the Piezo1 si-RNA, followed by treatment with 0% CMS or 10% CMS for 24 h after 8 h of serum starvation. **d** Representative Western blot images showing the activation of the PI3K-Akt-mTOR pathway. rMSCs were transfected with the Piezo1 si-RNA before they were subjected to 0% CMS or 10% CMS for 24 h after 8 h of serum starvation. **e** Quantitative RT-PCR was used to measure the expression of miR-337 in rMSCs treated with DMSO or Yoda1 and transfected with the miR-337 mimic as indicated simultaneously, followed by treatment with 0% CMS or 10% CMS for 24 h after 8 h of serum starvation. **f** Flow cytometry analysis showing the percentage of EdU^+^ rMSCs after treatment with DMSO or Yoda1 and transfection with the miR-337 mimic as indicated simultaneously, followed by treatment with 0% CMS or 10% CMS for 24 h after 8 h of serum starvation. **g** Representative Western blot images showing the activation of the PI3K-Akt-mTOR pathway. rMSCs were treated with DMSO or Yoda1 and simultaneously transfected with the miR-337 mimic as indicated, followed by treatment with 0% CMS or 10% CMS for 24 h after 8 h of serum starvation. **h** Schematic illustration of the TEAD binding site in the wild-type and mutant miR-337 promoters. The TEAD binding motif in the promoter of miR-337 was predicted using the JASPAR database. **i** Fluorescence activity in 293 T cells expressing the miR-337 wild-type or mutant promoter with TEAD pcDNA3.1. **j** Representative Western blot images showing the YAP protein level at 72 h after siRNA transfection. **k** Quantitative RT-PCR analysis of the expression of miR-337 in rMSCs treated with DMSO or Yoda1 and transfected with the YAP si-RNA simultaneously for 24 h after 8 h of serum starvation. **l** Quantitative RT-PCR analysis of the expression of miR-337 in rMSCs treated with the indicated medium for 24 h after 8 h of serum starvation. Statistical significance was assessed by Student’s *t*-test of the results from 3 independent experiments. *n* = 3 per group. *P < 0.05, **P < 0.01, and ***P < 0.001. The data are presented as means ± SD. The miR-337 expression levels were normalized to those of U6.

Piezo1 has been shown to function as a hydrostatic pressure receptor in MSCs, promoting the osteogenic differentiation of MSCs and inhibiting adipocyte differentiation^55^. We hypothesize that Piezo1 may be a key receptor through which MSCs sense extracellular mechanical signals and then convert them into extracellular biochemical signals. After siRNAs were used to knock down Piezo1 expression, the miR-337 expression level did not decrease in MSCs following exposure to 10% CMS, and 10% CMS also failed to promote the proliferation of MSCs or activate the PI3K-Akt signaling pathway, suggesting that miR-337 may be one of the downstream intracellular signal transduction targets of Piezo1 (Fig. 5a–d). After quiescent MSCs were treated with Yoda1, a small molecule activator of Piezo1, the expression level of miR-337 decreased, the proliferative ability increased, and the PI3K-Akt signaling pathway was activated (Fig. 5e–g). Conversely, following the overexpression of miR-337 in MSCs, the proliferation-promoting ability of Yoda1 was significantly attenuated, which corresponded with the sustained inhibition of the PI3K-Akt signaling pathway (Fig. 5e–g). The Hippo-YAP signaling pathway is one of the classic signaling pathways downstream of Piezo1^42^. We hypothesize that it may be a downstream pathway through which Piezo1 regulates miR-337 expression. Piezo1 activation can induce YAP nuclear translocation, and YAP typically acts as a transcriptional coregulator, forming a complex with TEAD to bind gene promoters and regulate downstream target gene expression^56–59^. We performed a bioinformatic analysis to predict TEAD binding motifs in the miR-337 promoter region (Fig. 5h). Dual-luciferase reporter assays further demonstrated the direct binding of TEAD to the promoter region (Fig. 5i). After the use of a small interfering RNA or a specific YAP inhibitor, verteporfin, to knock down or interfere with the YAP protein, the decrease in miR-337 expression induced by Yoda1 treatment no longer occurred (Fig. 5j–l). Collectively, these data suggest that MSCs sense extracellular mechanical signals through Piezo1 and regulate MSC proliferation by targeting the PI3K-Akt signaling pathway, which occurs at least in part through the downregulation of miR-337 by the Hippo-YAP signaling pathway.

### Knockout of miR-337 rescues HU-induced bone loss

**Fig. 6:**
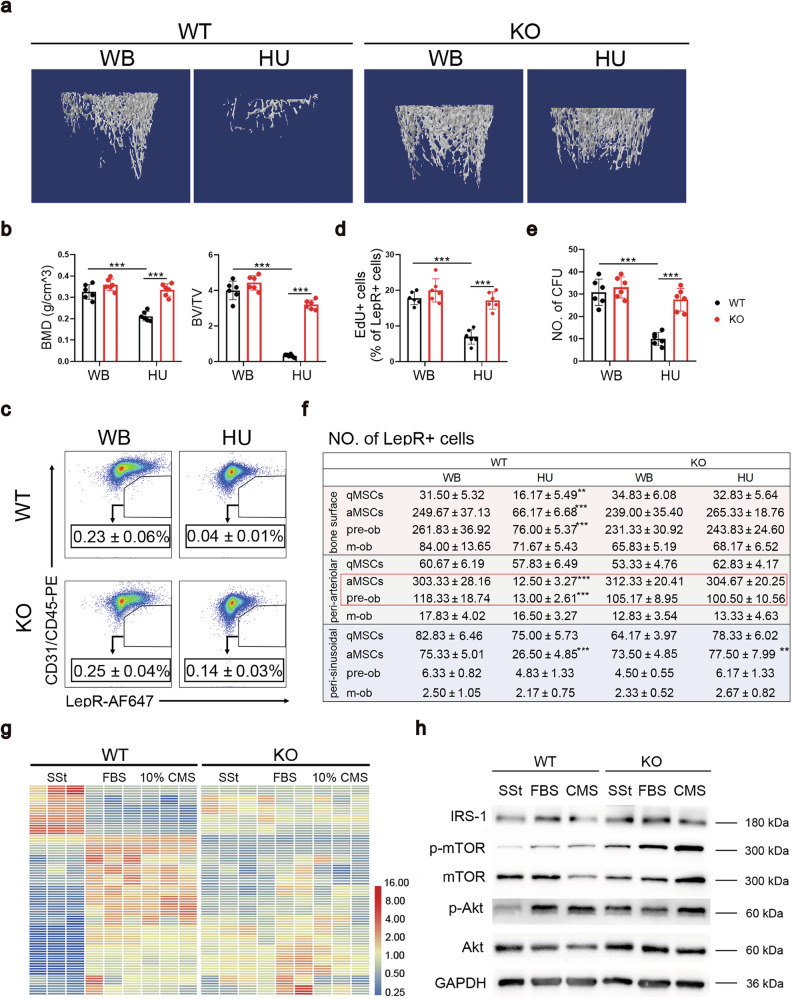
Knockout of miR-337 reversed mechanical unloading-induced bone loss. **a** Representative images of micro-CT and bone histomorphometry. WT and miR-337^–^^/–^ rats were suspended by the tail for 28 days before the femurs were used for micro-CT scanning. *n* = 6 per group. **b** Quantification of the three-dimensional microstructural parameters from micro-CT scans of femurs. **c** Representative images showing the flow cytometry analysis of CD45/CD31^–^LepR^+^ cells in the BM of WT and miR-337^–^^/–^ rats suspended for 7 days. **d** An EdU incorporation assay was used to determine the percentage of proliferative LepR^+^ cells in the BM of WT- or KO-rats after being suspended for 7 days. **e** CFU frequencies of BM cells isolated from WT- or KO-rats after being suspended for 7 days. Colonies larger than 40 cells were counted. **f** The number of subsets of LepR^+^ cells counted in 3 BM niches (< 50 μm) from mIHC images. **g** Heatmap of 21 DEGs enriched in the PI3K-Akt pathway, whose expression changed after 10% CMS in wild-type MSCs, but did not change significantly in KO-MSCs stimulated with FBS or 10% CMS. **h** Representative images of Western blots showing PI3K-Akt-mTOR pathway activity in WT- and miR-337^–^^/–^ MSCs after serum starvation for 12 h. Statistical significance was assessed by Student’s *t*-test of *t*he results from 3 to 6 independent experiments. All the data are presented as the means ± SD. *P < 0.05, **P < 0.01, and ***P < 0.001.

We generated miR-337 knockout (KO) rats to further confirm that the mechanical unloading-induced decreases in MSC contents were mediated, at least in part, by miR-337, (Supplementary Fig. S2j). Bone histological and morphological analyses showed that bone tissue and the remodeling balance were not affected in adult KO rats compared with WT littermates (Supplementary Fig. S3a–d), nor were the MSC CFU frequencies (Supplementary Fig. S3e). Remarkably, miR-337 KO greatly attenuated HU-induced bone loss (Fig. 6a, b) and the loss of LepR^+^ MSCs (Fig. 6c–e and Supplementary Fig. S4c, d). Moreover, bone formation following HU was substantially increased in KO rats compared with WT animals (Supplementary Fig. S4a, b). We further investigated the effect of miR-337 on MSC subsets by analyzing the frequencies of four subsets of LepR^+^ cells in three different niche locations in the BM of KO rats following HU. Unlike those in WT rats, the frequencies of aMSCs and pre-obs were not decreased in all three niche locations (bone surface, arterioles, and sinusoids) upon HU in KO rats, suggesting a significant attenuation of HU-induced bone loss (Fig. 6f and Supplementary Fig. S5a). Taken together, these data demonstrate that the inhibition of a single miRNA, miR-337, is sufficient to rescue disuse-induced bone loss in vivo.

We compared transcriptomic changes in cultured MSCs derived from WT and miR-337-KO rats subjected to SSt, FBS, or 10% CMS treatment. Compared with those in the SSt groups, genes enriched in the PI3K-Akt pathway were significantly altered in WT-MSCs after treatment with FBS or 10% CMS. However, these changes were essentially eliminated in the KO-MSCs (Fig. 6g). Consistent with the finding that miR-337 functions as an inhibitor of IRS-1 protein expression, higher IRS-1 protein levels corresponded to increased PI3K-Akt pathway activation (Fig. 6h). We detected elevated levels of phosphorylated Akt and mTOR in miR-337-KO MSCs 24 h after SSt than in WT MSCs (Fig. 6h), indicating the constitutive activation of the PI3K-Akt-mTOR pathway. Rapamycin treatment also reversed the protective effect of miR-337-KO on HU-induced bone loss (Supplementary Fig. S5b, c), further indicating that miR-337-KO rescues HU-induced bone loss through the activation of the PI3K-Akt-mTOR pathway.

### miR-337-KO MSCs function as therapeutic agents for antagonizing HU-induced bone loss

**Fig. 7:**
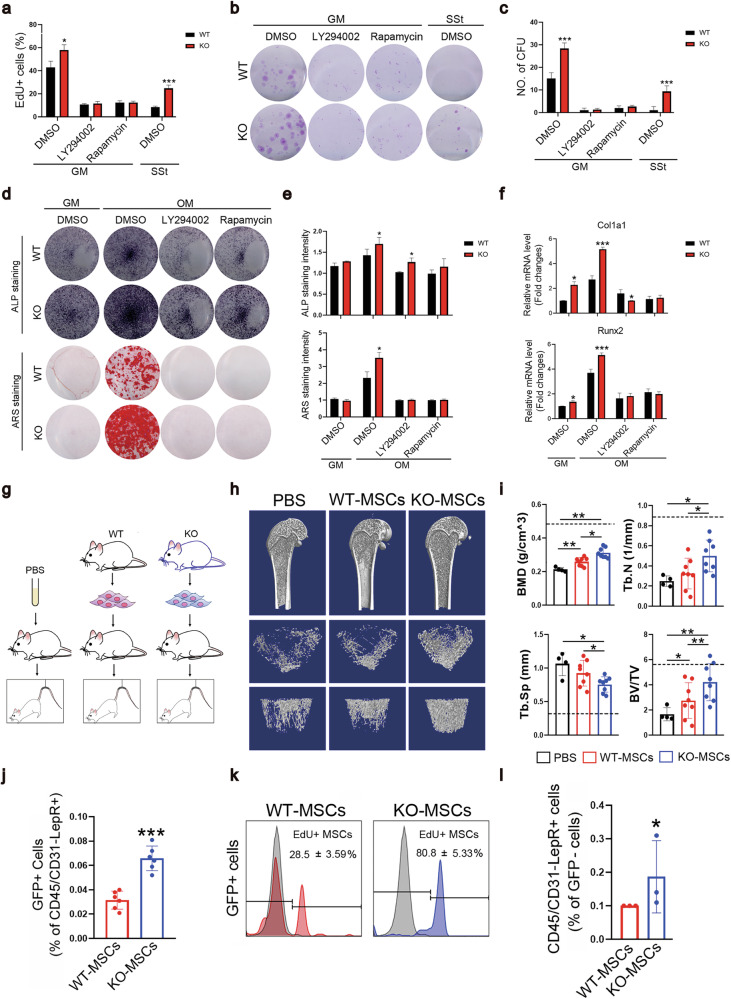
miR-337^–^^/–^ MSCs ameliorated the HU-induced decrease in bone formation. **a** Flow cytometry analysis of EdU^+^ MSCs treated as indicated for 24 h. **b** Representative images of the CFU frequency of WT- and miR-337^–/–^ MSCs in the growth medium either without serum or supplemented with a PI3K inhibitor (LY) or an mTOR inhibitor (Rapa). **c** The number of colonies containing more than 40 cells was quantified. **d** Representative images of ALP staining and ARS staining of WT- and miR-337^–/–^ MSCs treated as indicated. **e** Quantification of the staining intensity in three independent experiments. **f** Relative expression of Runx2 and Col1a1 in WT- and miR-337^–/–^ MSCs treated as indicated for 7 days. The data are normalized to the level of GAPDH. **g** Schematic of the in vivo transplantation assays. **h** Representative micro-CT images of femurs from WT rats suspended for 28 days. **i** Quantification of three-dimensional microstructural parameters from micro-CT scans of femurs. The baselines (black dotted lines) indicate the parameters of WT rats under WB conditions. **j** Flow cytometry analysis of transplanted GFP^+^ cells in the BM of WT rats suspended for 7 days. **k** Representative images of EdU^+^GFP^+^ cells in the BM of WT rats suspended for 7 days. **l** Flow cytometry analysis of endogenous CD45/CD31^–^LepR^+^ cells in the BM of WT rats transplanted with the indicated cells before 7 days of HU. The statistical significance of the results shown in **(a**–**f** and **j**–**l)** was assessed by Student’s *t*-test of 3 to 6 independent experiments. The data are represented as the means ± SDs. The statistical significance of the results from the experiments shown in i was assessed by one-way ANOVA with Dunnett’s post hoc test. *n* = 6 per group. The data are presented as means ± SEM. *P < 0.05, **P < 0.01, and ***P < 0.001.

Compared with WT MSC cultures, KO MSC cultures contained significantly more EdU-positive proliferating cells under SSt conditions (Fig. 7a), and the proliferation of WT cells in growth medium was PI3K-Akt-mTOR dependent, which was inhibited by LY294002 (a PI3K inhibitor) or rapamycin (an mTOR inhibitor). miR-337-KO MSCs also presented an increased colony formation capacity (Fig. 7b, c), and osteoblastic differentiation potential (Fig. 7d–f), which were also abrogated by LY294002 or rapamycin. The effects of miR-337 were specific to disuse-induced bone loss because miR-337 KO failed to prevent bone loss in rats after ovariectomy (OVX) (Supplementary Fig. S6a, b). Thus, different mechanisms are involved in estrogen deficiency-induced bone loss than in disuse-induced bone loss. OVX-treated rats exhibited significant bone resorption, but bone formation was only slightly increased, similar to previous observations^60,61^. OVX-induced bone remodeling changes occurred similarly in WT and miR-337 KO rats (Supplementary Fig. S6c, d), further suggesting that miR-337 does not play a role in OVX-induced bone loss.

Given the ability of miR-337 KO to potently protect against HU-induced bone loss in vivo, we sought to develop a more clinically applicable approach. We tested the possibility of using miR-337-KO MSCs alone to treat HU-induced bone loss in WT rats. We labeled MSCs derived from either miR-337-KO rats or WT littermates and intravenously injected WT rats with 10^5^ GFP-MSCs (in 100 μL of saline) through the tail vein, immediately before tail suspension (Fig. 7g). Remarkably, 28 days after suspension, the bone volume and BMD of the rats treated with a single administration of KO-MSCs were higher (Fig. 7h, i) than those of the uninjected animals. Compared with KO-MSCs, WT-MSCs improved bone mass retention under HU conditions, but were less effective (Fig. 7h, i). By 7 days of HU, transplanted GFP^+^ MSCs (WT vs. KO) were still present in the BM (Fig. 7j), but the number of LepR^+^ exogenous KO-MSCs was nearly twice that of exogenous WT-MSCs (Fig. 7j). Moreover, in unloaded BM, more than 80% of KO-MSCs were EdU positive at 7 days after suspension (Fig. 7k), indicating a higher proliferation rate than that of WT-MSCs. Interestingly, the number of GFP^–^ endogenous LepR^+^ MSCs also increased after HU following the transplantation of KO-MSCs, suggesting the presence of noncell-autonomous effects (Fig. 7l).

These results demonstrate that targeting miR-337 in MSCs maintains their ability to proliferate and differentiate into osteogenic lineage cells in the pathogenic BM microenvironment induced by HU and that supplementation with exogenous KO-MSCs not only provides stronger “seeds” for bone formation but also modulates the microenvironment “soil” of the BM to attenuate pathological damage to endogenous stem cells.

## Discussion

In this study, we found that the frequency of proliferating LepR^+^ cells decreased in the BM of rodents subjected to HU, which is one of the main reasons for decreased bone formation. As osteoblasts are short-lived cells that need constant supplementation from a proliferating pool to maintain their number and new bone formation throughout life, our results provide evidence that the loss of proliferating LepR^+^ cells is the main reason for decreased bone regeneration in disuse-induced osteoporosis.

BM MSCs display extraordinary heterogeneity and diverse functions depending on their ontogeny and local niche. Identifying the changes in specific MSC subsets under different conditions allows us to define functional changes in certain disease situations. We noticed that only the frequency of proliferating LepR^+^ cells was changed in a rodent model of disuse-induced bone loss. We hypothesize that mechanical unloading arrests the proliferation of subsets of LepR^+^ cells and causes cell cycle exit. Proliferating LepR^+^ cells reside in the metaphysis (neck portion of a long bone). Here, preosteoblasts (Runx2^+^) are closer to the surface, whereas Runx2^–^ cells are closer to the arterioles. BM arterioles are reported to be one of the key MSC niches that couple bone formation, while peri-arteriolar LepR^+^ cells are the main source of skeletal stem cells for bone regeneration^62^. Therefore, the speculation that quiescent MSCs are activated at the peri-arteriolar niche to proliferate and enter an osteogenic program while migrating to the bone-forming surface is reasonable. Although many cells undergo apoptosis during this process, some can finally reach the bone surface and become mature bone lining cells to secrete bone matrix for new bone formation.

Consistent with the above hypothesis, arterioles, not sinusoids, constitute the main niche for MSC activation, as many fewer proliferating LepR^+^ cells are located around sinusoids. The peri-arteriolar region represents a “stronger” mechanical environment because the stiffer and thicker arteriolar wall is more effective at transmitting movement-induced mechanical stimuli than the sinusoidal wall^25,63^. In contrast, LepR^+^ cells are maintained in a quiescent state by the “weaker” mechanical environment of the perisinusoidal niche. Although vascular endothelial cells are likely to secrete cytokines to maintain the proliferation of MSCs, our RNA-seq analysis revealed that mechanical stretching stimulates qMSCs to enter the cell cycle by the same mechanism as growth factors. The next challenge is to measure the mechanical strength transmitted via BM arterioles and sinusoids under both physiological and pathological conditions. We cannot conclude that the activation of qMSCs at the peri-arteriolar niche is completely induced by mechanical stretching. Growth factors secreted from local niche cells might also have certain contributions. However, our results indicate that the peri-arteriolar niche is a mechanosensitive MSC niches that affect new bone formation in addition to the previously reported endosteal niche. A picture of the BM microenvironment under HU conditions emerges. Without mechanical unloading, arterioles become thinner and less effective at transmitting mechanical signals and activating local qMSCs. These events lead to a decreased frequency of LepR^+^ cells and ultimately the inhibition of bone regeneration. Activating LepR^+^ cells in pathological BM could be a new strategy to compensate for mechanical unloading-induced bone loss, and targeting the PI3K-Akt pathway could also augment this strategy.

The PI3K-Akt signaling pathway has been reported to be one of the key regulators of the activation of multiple adult stem cells, including HSCs, NSCs, and satellite cells. Given that PI3K-Akt are the main targets of miR-337, we hypothesize that the function of this miRNA is to maintain stem cells in a quiescent state. The expression level of miR-337 in LepR^+^ cells was much higher than that in control cells, which was consistent with their quiescent state in unloaded BM. Moreover, LepR^+^ cells derived from miR-337^–^^/–^ rats maintained their proliferation and osteogenic differentiation in unloaded BM, indicating that miR-337 is the main target for regulating the activity of LepR^+^ cells. Our RNA-seq data also revealed additional microRNAs with expression patterns similar to those of miR-337 in mechanically stimulated cellular models. To our knowledge, these miRNAs have not been previously associated with mechanical stress responses in the literature and thus represent novel and uncharacterized components in mechanical stress transduction pathways; thus, future studies are needed to explore their roles and mechanisms further.

**Fig. 8:**
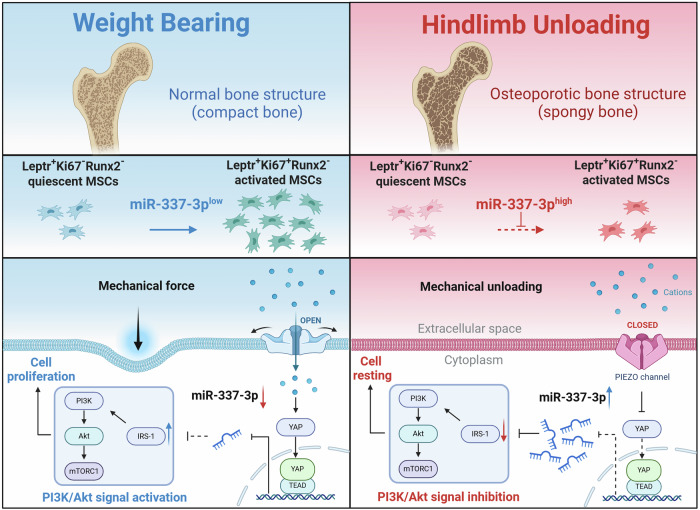
Schematic diagram illustrating the regulation of mesenchymal stem cell proliferation by mechanical stress via miR-337 expression. HU results in the loss of mechanical stress around quiescent LepR^+^ mesenchymal stem cells, leading to the closure of the Piezo1 channel. Through the YAP-TEAD pathway, this closure increases the expression of miR-337, which inhibits the PI3K-Akt-mTOR signaling pathway. Consequently, the number of activated LepR^+^ MSCs in adult BM decreases, contributing to bone loss.

Piezo1 has been reported to function as a hydrostatic pressure receptor in MSCs, promoting the osteogenic differentiation of MSCs and inhibiting adipocyte differentiation^55^. The Hippo-YAP signaling pathway is one of its classic downstream signaling pathways^42^. Here, we speculate that Piezo1, which senses extracellular mechanical signals, may regulate the proliferation of MSCs by targeting the PI3K-Akt signaling pathway by the downregulation of miR-337 through the Hippo-YAP signaling pathway. Dual-luciferase reporter assays and functional studies demonstrated that YAP/TEAD bind to the promoter region of miR-337 and suppress its expression. After treatment with 10% CMS or Yoda1, miR-337 expression decreased, the PI3K-Akt signaling pathway was activated, and the proliferative ability of MSCs was significantly improved. This phenomenon disappeared after the use of siRNAs to knock down Piezo1 or mimics to overexpress miR-337, indicating that MSCs sense extracellular mechanical signals through Piezo1 and regulate the proliferation of MSCs by targeting the PI3K-Akt signaling pathway through the suppression of miR-337 expression (Fig. 8).

Our study revealed that HU-induced MSC depletion is driven primarily by suppressed proliferation caused by high miR-337 expression. However, these LepR^+^ cells may gradually undergo apoptosis after exiting the cell cycle. We cannot rule out the possibility that mechanical unloading stimulates more severe apoptosis in LepR^+^ cells. Recent evidence has suggested that apoptosis-senescence dynamics may also synergistically exacerbate MSC loss under mechanical unloading^64^. The HU-induced downregulation of Piezo1 could also reduce the production of apoptotic vesicles, impairing senescent cell clearance and promoting MSC dysfunction. Senescent MSCs not only lose their regenerative capacity but also secrete proinflammatory factors that further suppress neighboring MSC activity. Our data showed that miR-337 overexpression or knock down did not alter MSC apoptosis or senescence markers expression (Supplementary Fig. S7a–h). Its proliferation-specific effects likely operate in parallel with pathways mediating apoptosis resistance or senescence accumulation, collectively compromising MSC pools. Our work advances the understanding of HU-related MSC depletion by identifying a miR-337-regulated proliferative pathway, while underscoring the need to evaluate the crosstalk between proliferation, apoptosis, and senescence in future therapeutic strategies targeting mechanical unloading pathologies.

In addition to its roles in stress transduction and proliferation modulation, miR-337 has the potential to regulate MSC osteogenic differentiation, as evidenced by the reduced ALP staining intensity upon miR-337 overexpression and enhanced mineralization following its knockdown. Our previous study demonstrated that miR-337 mediated osteogenic and chondrogenic differentiation in tendon-derived stem cells by the IRS-1/ERK/Runx2 and NOX4/JNK/Sox9 pathways, respectively^36^. Regarding adipogenic differentiation, qPCR analyses revealed no significant changes in adipogenesis marker expression upon either miR-337 knockdown or overexpression (Supplementary Fig. S7i–k), indicating that miR-337 is a selective regulator of the osteochondral differentiation of MSCs. Osteoclastogenic markers also remained unaffected by miR-337 manipulation (Supplementary Fig. S7l–p), despite the enhanced bone resorption observed in miR-337 knockout rats subjected to HU. This apparent discrepancy may arise from compensatory coupling between augmented osteogenesis and physiological remodeling processes, as bone formation and resorption frequently coordinate during skeletal adaptation. Parallel investigations by our team specifically explored the contributions of osteoclast precursors to unloading-induced bone loss (data not shown).

Additionally, the non-significant impact of miR-337 KO on bone loss induced by OVX may reflect the distinct mechanistic pathways through which miR-337 and estrogen deficiency regulate bone homeostasis. OVX primarily drives bone loss via estrogen withdrawal, which disrupts the balance between bone resorption and formation. This process is predominantly mediated by upregulated pro-osteoclastic cytokines, including RANKL and TNF-α, and impaired osteoblast activity due to estrogen receptor signaling deficits^65,66^. Our data suggest that miR-337 may not intersect with the estrogen-depletion axis critical to OVX pathology.

As MSCs are the main niche cells that maintain the frequency and functions of HSCs, inadequate MSCs in unloaded BM might also affect lymphopoiesis and myogenesis, leading to decreased immunity and increased systemic inflammation^25,67,68^. Therefore, supplementation with exogenous miR-337^–^^/–^ MSCs, which can resist the pathological BM microenvironment, might be an effective way to alleviate the bone loss and systemic immune dysregulation induced by mechanical unloading. Given that miR-337 is regulated similarly in human BM-derived MSCs and rat MSCs, we speculate that our findings might be translatable to humans. Together, these findings have implications for both long-term spaceflight-induced bone loss and disuse-induced bone loss observed in bedridden patients.

Several limitations of this study should be acknowledged. First, the use of the HU model to study prolonged mechanical unloading was inherently limited to 30 days due to severe complications, including heightened aggression, self-mutilation, and tail necrosis caused by abrasion-induced ischemia, which not only raises ethical concerns regarding animal welfare but also compromises data reliability beyond this timeframe. Consequently, the long-term effects of miR-337 KO or miR-337-modulated stem cell therapy on disuse-induced bone loss remain unexplored. Second, a biomechanical discrepancy exists between the current experimental models and actual microgravity conditions. Future studies may require validation through real-space experiments. Additionally, the therapeutic potential of miR-337 KO stem cells remains limited in clinical translation because of challenges in scalability and safety. While no tumorigenic effects were observed in our current animal experiments, future preclinical evaluations must rigorously assess the safety risks associated with sustained PI3K-Akt pathway activation, given its oncogenic potential. Further development of clinically translatable, targeted strategies to mitigate these risks remains critical to enhance clinical relevance.

## Materials and methods

### Animals and HU model

Two-month-old male Sprague-Dawley rats were purchased from Shanghai SLAC Laboratory Animal Co., Ltd (Shanghai, China) and were caged under standard conditions. miR-337 KO rats were generated by Cyagen Corporation, China. miR-337 KO animals were genotyped by PCR with the following primer sequence, forward, 5’-GGTCCCAGTGTAGTGAGAAGTT-3’, and reverse, 5-CGTCCCTAAGCAGTCCAAG-3’.

For HU, a strip of medical adhesion tape was applied along with the proximal one-third of the tail and attached to a metal bar on the tip of the cage. The suspension height was adjusted to prevent the hindlimbs from touching any supporting surface while the forelimbs were able to contact the grid floor for free movement. All the experimental procedures were approved by the Committees of Animal Ethics and Experimental Safety of Shanghai Jiaotong University.

### Micro-CT analysis and bone histomorphometry

The femurs and tibiae were collected, and the attached soft tissue was removed before fixation with 4% paraformaldehyde. The fixed femurs were scanned using a SkyScan1076 (Bruker micro-CT, Kontich, Belgium). A voxel size of 12 µm was chosen in all three spatial dimensions. For each sample, 148 of the 232 slices were evaluated covering a total of 1.776 mm at a voltage of 70k Vp, an intensity 114 µA, and an integration time of 1200 ms. Three-dimensional models of the trabecular bone of the femur were reconstructed using SkyScan CT Analyzer version 1.17 to evaluate the alterations in bone and the structural parameters.

For the bone remodeling analysis, trabecular sections were subjected to tartrate-resistant acid phosphatase (TRAP) staining and ALP staining with commercial kits (Beyotime Biotechnology, Shanghai, China) according to the manufacturer’s instructions.

### mIHC analysis

Tibia sections were used for the TSA Opal mIHC analysis according to the manufacturer’s instructions (Opal PerkinElmer). Briefly, tibia sections were deparaffinized and heat-induced epitope retrieval (HIER) was performed for 20 min at 95 °C in pH 6.0 citrate buffer after blocking endogenous peroxidase in tissues with 0.3% H_2_O_2_/methanol for 10 min. The sections were washed with PBS 3 times and blocked with blocking buffer for 10 min, before an incubation with a primary antibody for 1 h. Next, the tissue sections were washed 2 times with Tris-buffered saline-0.05% Tween 20 (TBST) and incubated with horseradish peroxidase (HRP)-conjugated anti-mouse or anti-rabbit secondary antibody for 10 min. After washing, the tibia sections were incubated with an Opal fluorophore for 10 min. Bound primary and secondary antibodies were then eluted with HIER treatment. After washing with PBS, the process of staining and antibody removal was repeated using different Opal fluorophores until all the markers were stained. Finally, the tissue sections were stained with 4′,6-diamidino-2-phenylindole (DAPI) for 5 min and mounted in ProLong Diamond Antifade Mountant (Thermo Fisher Scientific) Dako medium. A Vectro Polaris automated quantitative pathology imaging system (Akoya Biosciences) was used for multispectral imaging at 20× magnification. Whole slide images were analyzed with InForm image analysis software (Akoya Bioscience).

### Isolation of MSCs and CMS application

MSC isolation and in vitro culture were performed as previously reported. Briefly, the BM cells were flushed out from the femurs and tibiae with growth medium (α-MEM supplemented with 10% FBS) into 10 cm dishes. The cells were allowed to adhere and grow for 3 days before the GM was replaced, after which they continued to grow until they reached confluence. The cells were not used beyond P5. For CMS application, MSCs were seeded on six-well BioFlex culture plates coated with collagen type I (Flexcell International Corporation, Hillsborough, NC, USA). CMS with a 0.5 Hz sinusoidal curve at the indicated elongation was applied (80,000 με, Sin, 0.5 Hz, CMS) using an FX5000T Flexcell Tension Plus unit (Flexcell International Corporation). The cells were incubated in a humidified atmosphere at 37 °C with 5% CO_2_ while they were stretched. The cells were harvested immediately after the completion of CMS stimulation.

### RNA sequencing

For the experiment shown in Fig. 2, MSCs were seeded on six-well BioFlex culture plates and cultured in GM for 12 h to allow them to adhere to the plates. Afterward, the cells were serum-starved for 8 h before CMS stimulation or serum supplementation for another 16 h. Total RNA was collected using the TRIzol extraction method, and sample quality was assessed using a Nanodrop 2000 spectrophotometer (Thermo Scientific). RNA sequencing was performed by LC-Bio Technology Co., Ltd., Hangzhou, China.

### Flow cytometry

For the flow cytometry analysis, BM cells were flushed from tibiae and femurs with PBS/2% FBS and collected by centrifugation at 300x *g* for 10 min. After red blood cells were lysed with ACK buffer, an equal number of BM cells were subjected to immunostaining with the primary antibodies listed in Table S1 at 4 °C for 30 min, and then washed with FACS buffer. After being resuspended in 100 μL of FACS buffer, the cells were subjected to a FACS analysis with a Guaca easyCyte 12Base System (Millipore). The data were analyzed with FlowJo V10 software.

### In vivo transplantation

For the experiment shown in Fig. 6, MSCs were isolated from 2-month-old male miR-337-KO rats and WT littermates. Both WT- and KO-MSCs at P2 were stably transfected with lentiviruses expressing GFP. The GFP-MSCs were collected at P3 by trypsin digestion and resuspended in PBS at a density of 10^6^ cells per ml. One hundred microliters of cells were intravenously injected into 2-month-old male WT rats by the tail vein. Recipient rats were subjected to HU immediately after transplantation. At 5 days post-unloading, EdU was intraperitoneally injected, and rats were then suspended for another 2 days before being sacrificed on day 7. Total BM cells were prepared for the FACS analysis. In some cases, the recipient rats were suspended for 28 days for the bone histomorphological analysis.

### Ocn and CTX concentration

The sera of animal models were prepared from the blood of each animal by centrifugation at 2000x *g* for 10 min. A rat-specific Ocn ELISA kit (Biomedical Technologies, Inc.) and a rat-specific CTX I ELISA kit (Immunodiagnostic Systems Ltd.) were used.

### Quantitative RT-PCR

For the analysis of miRNA expression in MSCs, the BM cells from both WB- and HU-rats were prepared and stained with an anti-LepR antibody (Novus) for 30 min at 4 °C. After washing, cells were stained with PE-conjugated anti-rabbit secondary antibody for 15 min. After washing, the cells were incubated with anti-PE beads for 15 min at 4 °C and subjected to magnetic cell sorting as described above. The sorted LepR^+^ cells were collected and directly resuspended in TRIzol reagent, followed by RNA extraction. First-strand cDNA was synthesized from 1 μg of total RNA by incubation for 1 h at 42 °C with Superscript III reverse transcriptase (Invitrogen, Mulgrave, Australia) and specific miRNA RT primers. Quantitative RT-PCR was performed with a LightCycler480 system (Roche, Mannheim, Germany) using SYBR Premix Ex Taq^TM^ (Takara, Dalian, China) according to the manufacturer’s instructions. All amplifications were normalized to U6. The data were analyzed using the comparison Ct (2^–ΔΔCt^) method and reported as the fold changes compared with the respective control. Each sample was analyzed in triplicate. The primer sequences used in this study were purchased from RiboBio Co., Ltd (Guangzhou, China).

### Western blot

The cells were lysed with RIPA buffer supplemented with 1 mM phenylmethylsulfonyl fluoride, and protease inhibitor cocktail (10 mg/mL leupeptin, 10 mg/mL pepstatin A, and 10 mg/mL aprotinin) on ice for 30 min, followed by centrifugation at 15,000x *g* at 4 °C for 10 min. The total soluble protein in the supernatant was collected, and 20 μg of protein was resolved on 10% SDS-PAGE gels. Protein bands were transferred to polyvinylidene difluoride (PVDF) membranes (Whatman, Piscataway, NJ, USA). The membranes were blocked with 5% BSA and incubated with specific antibodies overnight at 4° C. After an incubation with a horseradish peroxidase-labeled secondary antibody for 1 h at RT, the immunoreactive bands were visualized using an enhanced chemiluminescence detection system (Millipore, Billerica, MA, USA) The immunoreactive bands were quantitatively analyzed in triplicate using ImageJ software, and the relative protein levels were calculated by normalizing the band intensities to those of GAPDH.

### CFU assay

A total of 1000 cultured MSCs or 10^6^ freshly extracted BM cells were seeded on a six-well plate and cultured between 6- and 14- days after plating. The cells were stained with crystal violet, and colonies larger than 50 cells were counted.

### EdU incorporation

For the in vitro proliferation assay, a commercial kit (RiboBio) was used according to the manufacturer’s protocols. Briefly, the EdU solution was diluted 1:1000 with culture medium and incubated with the MSCs for 2 h. Blank controls were treated with the same amount of DMSO. After the incubation, the cells were digested with trypsin and collected by centrifugation at 300x *g* for 10 min, followed by fixation with 4% PFA. After washing with PBS, the cells were permeabilized with 0.3% Triton X-100 for 10 min. The cells were washed twice with 3% BSA in PBS and then incubated with a freshly prepared Click-iT cocktail (Click-iT reaction buffer, CuSO4, Alexa Fluor 597 azide, and reaction buffer additive) for 30 min at RT. After washing, the cells were resuspended in PBS and subjected to FACS analysis.

For in vivo assays, 50 mg/kg EdU in DMSO was intraperitoneally injected 48 h prior to sacrifice. The BM cells were collected, and a click reaction was performed, as described above. In some cases, BM cells were stained with surface markers before the fixation step.

### Osteoblastic differentiation

MSCs at P3 were seeded onto a 12-well plate and cultured until reached 90% confluence. Afterward, the growth medium was replaced with osteogenic medium composed of 100 nM dexamethasone, 50 μM ascorbic acid, and 10 mM β-glycerophosphate. The cells were subjected to alkaline phosphatase staining 7 days after incubation, and Alizarin Red S staining 21 days after incubation, with the appropriate commercial kits (Beyotime Institute of Biotechnology, Shanghai, China).

### Dual luciferase reporter activity assay

The luciferase gene was cloned downstream of the wild-type or mutant promoter region of miR-337, and co-transfected into 293 T cells with the control vector and pcDNA3.1-TEAD. After 48 h, firefly luciferase activity was detected using the Dual-Luciferase® Reporter Assay System (Promega) and normalized to Renilla luciferase activity as the internal reference. The dual-luciferase assay was performed according to standard protocols (Promega, Madison, USA).

### Statistical analysis

Statistical analyses were performed using GraphPad Prism 8. The results are presented for individual rats and/or replicates as the means ± SEMs. The experimental rats are shown as individual dots. When more than 2 groups were compared, ANOVA was performed with multiple comparisons, reported in the figure legend. When only 2 groups were compared, an unpaired Student’s *t*-test was used, unless stated otherwise. Statistical significance is reported as the *P* value in the figure legend.

## Supplementary information

supplementary files

Supplementary tableS1

## Data Availability

Source data files are available from the corresponding author upon request. The RNA sequencing data have been submitted to the NCBI Gene Expression Omnibus (GEO) (accession number GSE 189845).
