# Supplementary material for: Targeting miR-337 mitigates disuse-induced bone loss
Source: Cell Discov. 2025 Aug 26;11:71. doi: 10.1038/s41421-025-00822-z (PMC12378244; doi:10.1038/s41421-025-00822-z)
Supplement: Supplementary file 1 — supplementary files [file 41421_2025_822_MOESM1_ESM.pdf]

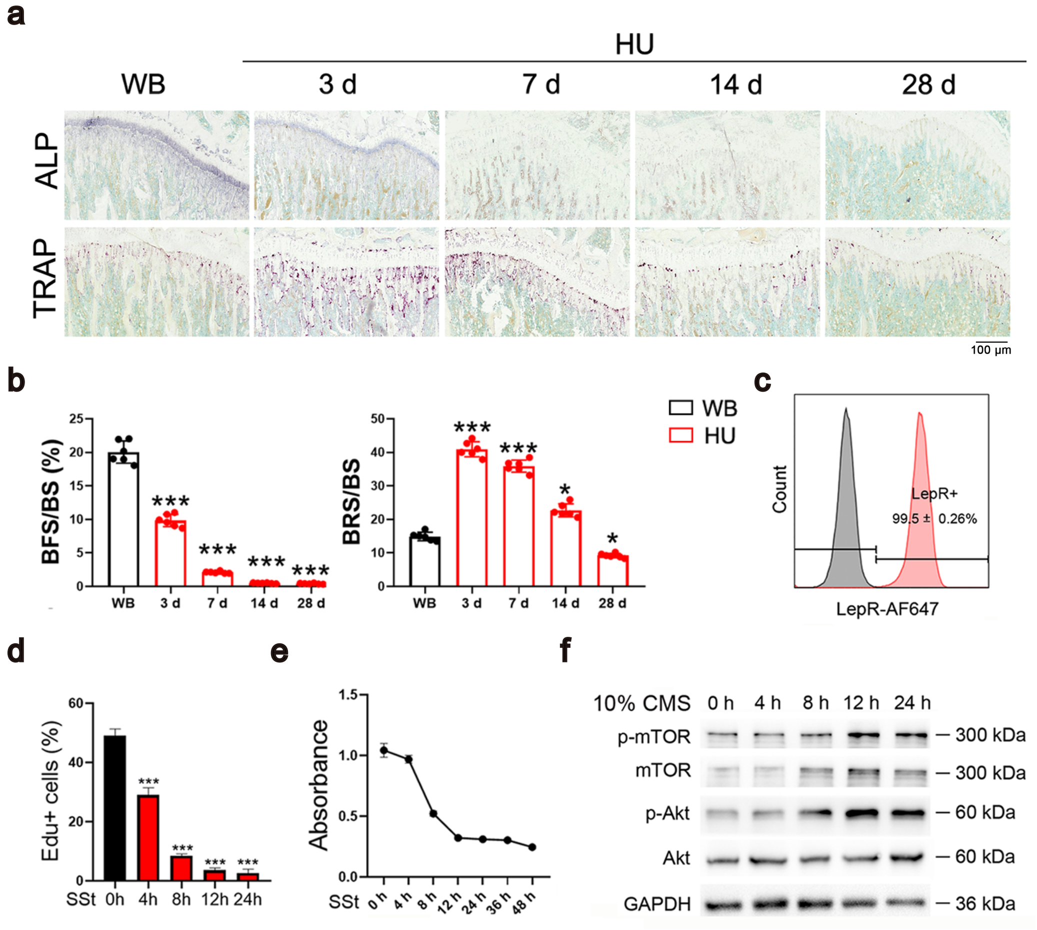
a
HU
WB
3 d
7 d
14 d
28 d
ALP
TRAP
100 μm
b
BFS/BS (%)
BRS/BS
WB
HU
c
Count
LepR+
99.5 ± 0.26%
LepR-AF647
d
Edu+ cells (%)
SSt 0h 4h 8h 12h 24h
e
Absorbance
SSt 0 h 4 h 8 h 12 h 24 h 36 h 48 h
f
10% CMS 0 h 4 h 8 h 12 h 24 h
p-mTOR — 300 kDa
mTOR — 300 kDa
p-Akt — 60 kDa
Akt — 60 kDa
GAPDH — 36 kDa

Supplementary Fig. S1 (a) Tibia sections from WB- and HU-treated rats were used for ALP (bone formation) and TRAP (bone resorption) staining. Scale bars, 100 μm. (b) Quantification of ALP and TRAP staining. The data are presented as percentages of the positively stained area relative to the total bone area. (c) Flow cytometry analysis of the positivity of LepR in in vitro cultured MSCs (P4). (d) Flow cytometry analysis of the percentage of EdU^+ MSCs after serum-starvation for the indicated durations. (e) CCK-8 assay showing the metabolite activities of MSCs after serum-starvation for the indicated durations. (f) Representative Western blot images showing the activation of the PI3K/Akt/mTOR pathway. The statistical significance of the results from the experiments shown in i-n was assessed by a two-tailed Student`s t-test. $^*P<0.05$, $^{**}P<0.01$, and $^{***}P<0.001$. The data are presented as means ± SDs of 3 independent experiments. The miR-337 expression levels were normalized to those of U6.

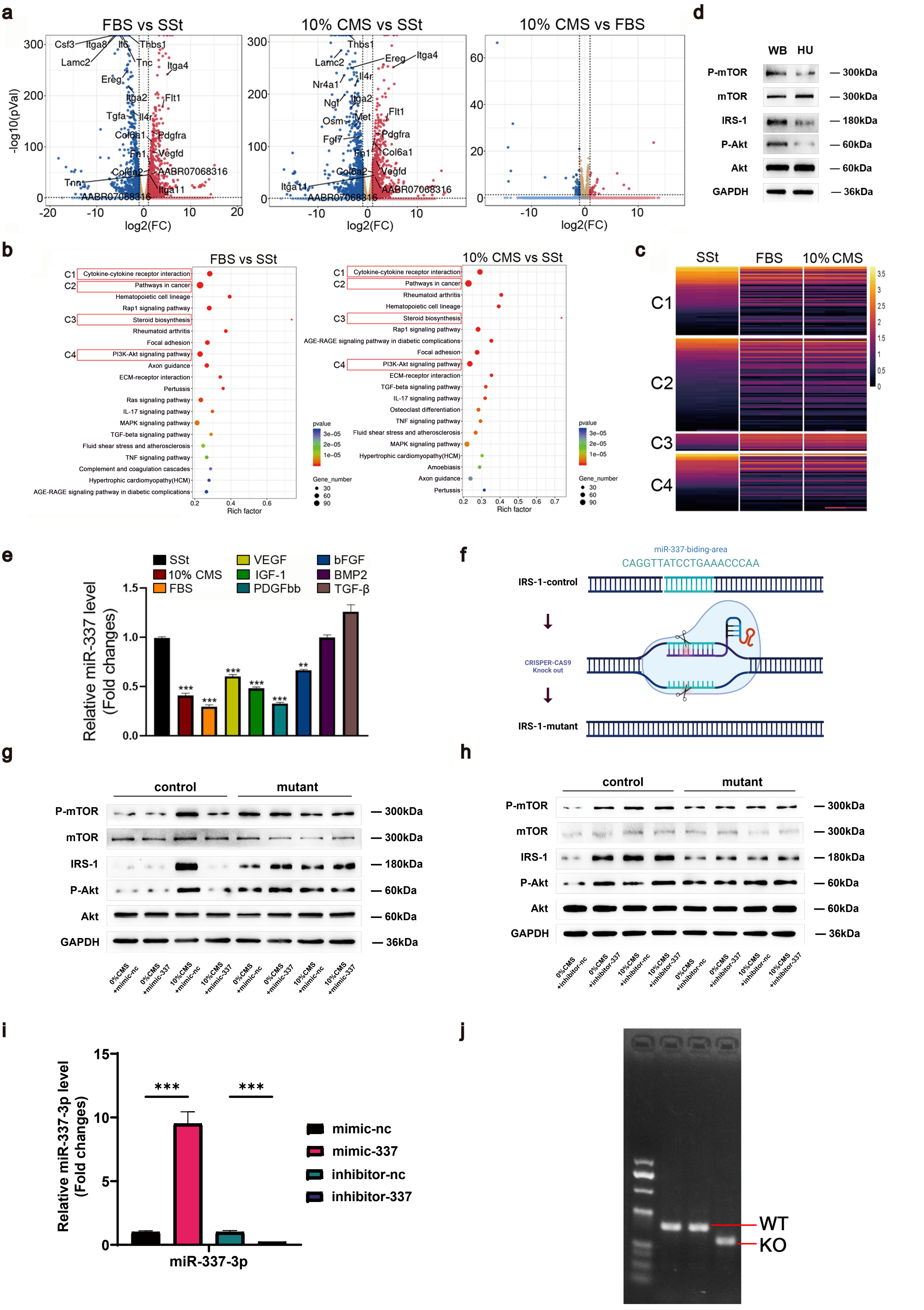
a
FBS vs SSt
10% CMS vs SSt
10% CMS vs FBS
-log10(pVal)
log2(FC)
Csf3
Itga8
Il6
Thbs1
Lamc2
Tnc
Itga4
Ereg
Itga2
Flt1
Tgfa
Il4r
Col6a1
Pdgfra
Fn1
Vegfd
Col6a2
AABR07068316
Tnn
Itga11
Nr4a1
Ngf
Osm
Met
Fgf7
d
WB
HU
P-mTOR
mTOR
IRS-1
P-Akt
Akt
GAPDH
— 300kDa
— 180kDa
— 60kDa
— 36kDa
b
FBS vs SSt
10% CMS vs SSt
C1 Cytokine-cytokine receptor interaction
C2 Pathways in cancer
Hematopoietic cell lineage
Rap1 signaling pathway
C3 Steroid biosynthesis
Rheumatoid arthritis
Focal adhesion
C4 PI3K-Akt signaling pathway
Axon guidance
ECM-receptor interaction
Pertussis
Ras signaling pathway
IL-17 signaling pathway
MAPK signaling pathway
TGF-beta signaling pathway
Fluid shear stress and atherosclerosis
TNF signaling pathway
Complement and coagulation cascades
Hypertrophic cardiomyopathy(HCM)
AGE-RAGE signaling pathway in diabetic complications
Osteoclast differentiation
Amoebiasis
pvalue
Gene_number
Rich factor
c
SSt
FBS
10% CMS
C1
C2
C3
C4
e
SSt
10% CMS
FBS
VEGF
IGF-1
PDGFbb
bFGF
BMP2
TGF-β
Relative miR-337 level (Fold changes)
f
miR-337-biding-area
CAGGTTATCCTGAAACCCAA
IRS-1-control
CRISPER-CAS9 Knock out
IRS-1-mutant
g
control
mutant
0%CMS +mimic-nc
0%CMS +mimic-337
10%CMS +mimic-nc
10%CMS +mimic-337
h
0%CMS +inhibitor-nc
0%CMS +inhibitor-337
10%CMS +inhibitor-nc
10%CMS +inhibitor-337
i
Relative miR-337-3p level (Fold changes)
mimic-nc
mimic-337
inhibitor-nc
inhibitor-337
miR-337-3p
j
WT
KO

Supplementary Fig. S2 (a) Volcano plot of differentially expressed genes (DEGs) identified by RNA-seq. The top 20 DEGs in the PI3K-Akt pathway are indicated. (b) The top 20 pathways enriched in cells treated with FBS or 10% CMS. (c) Heatmap of genes enriched in selected pathways. (d) Representative Western blot images showing the inactivation of the PI3K/Akt/mTOR pathway in de novo isolated LepR+ cells from rats suspended for 14 days and WB controls. (e) Quantitative RT-PCR was used to measure the expression of miR-337 in MSCs treated as indicated for 24 h after they were serum-starved for 8 h. (f) Schematic diagram of knockout of the miR-337 binding site in the IRS-1 gene using CRISPR-CAS9. (g-h) Representative Western blot images showing the activation of the PI3K-Akt-mTOR pathway. Con-rMSCs or mut-rMSCs were transfected with the indicated oligos, followed by treatment with 0% CMS or 10% CMS for 24 h after 8 h of serum starvation. (i) Quantitative RT-PCR was used to assess the overexpression and knockdown efficiency of miR-337 in human MSCs. (j) Genomic PCR and agarose gel electrophoresis results of wild-type (WT) and miR-337 knockout (KO) rats using primers flanking the targeted genomic region of miR-337. Statistical significance was assessed by Student`s t-test of 3 independent experiments. *$P<0.05$, **$P<0.01$, and ***$P<0.001$. The data are presented as means ± SDs. The miR-337 expression levels were normalized to those of U6.

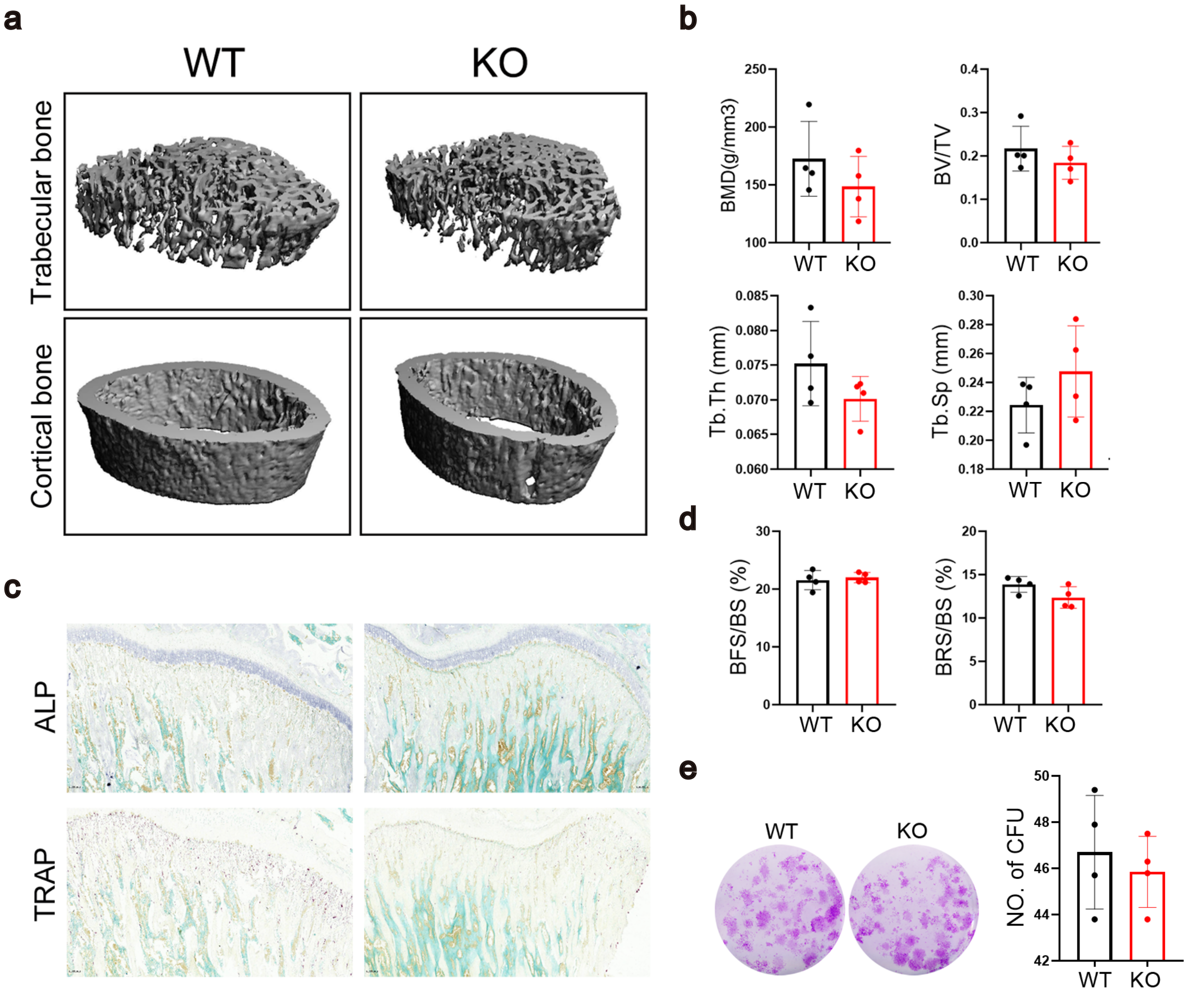

a
WT
KO
Trabecular bone
Cortical bone
b
BMD(g/mm3)
250
200
150
100
WT KO
BV/TV
0.4
0.3
0.2
0.1
0.0
WT KO
Tb.Th (mm)
0.085
0.080
0.075
0.070
0.065
0.060
WT KO
Tb.Sp (mm)
0.30
0.28
0.26
0.24
0.22
0.20
0.18
WT KO
c
ALP
TRAP
d
BFS/BS (%)
30
20
10
0
WT KO
BRS/BS (%)
20
15
10
5
0
WT KO
e
WT
KO
NO. of CFU
50
48
46
44
42
WT KO

Supplementary Fig. S3 (a) Representative micro-CT images showing the microarchitecture of trabecular and cortical bones of femurs from 2-month-old male miR-337-KO rats and their littermates. (b) Quantification of three-dimensional parameters analyzed by micro-CT scanning. n=4 per group. (c) Representative images of ALP and TRAP staining of tibia sections prepared from male 2-month-old miR-337-KO rats and their littermates. (d) Quantification of ALP (bone formation surface/bone surface: BFS/BS) and TRAP (bone resorption surface/bone surface: BRS/BS) staining. The data are presented as percentages of positively stained area relative to the total bone area. n=4 per group. (e) Representative images of CFU-F (left panel) and quantification of the numbers of colonies larger than 40 cells (right panel). Statistical significance was assessed by Student`s t-test of the results from 3 independent experiments. The data are presented as means ± SDs. n=4.

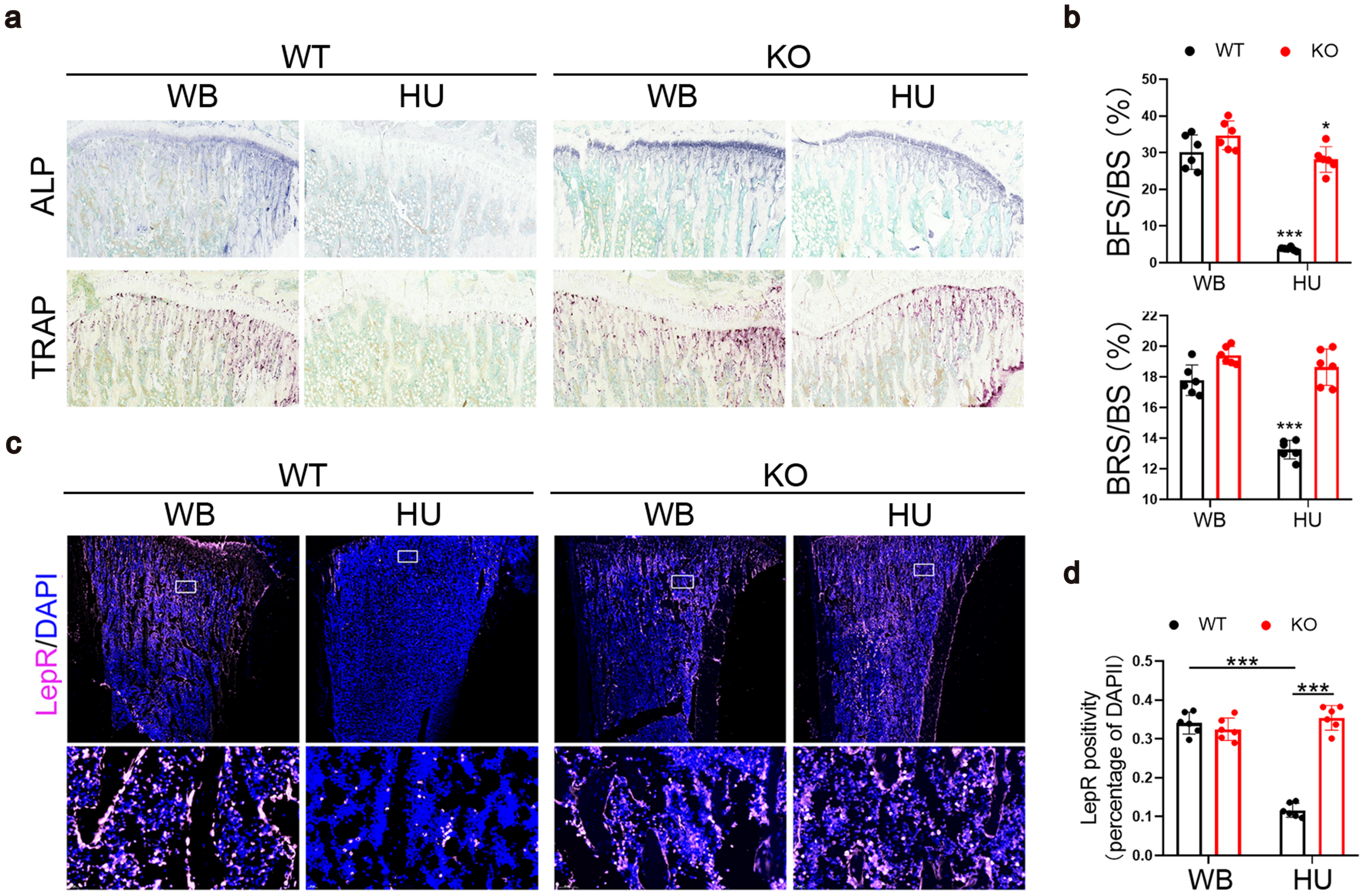
a
WT
KO
WB
HU
WB
HU
ALP
TRAP
b
WT
KO
BFS/BS（%）
50
40
30
20
10
0
***
*
WB
HU
BRS/BS（%）
22
20
18
16
14
12
10
***
WB
HU
c
WT
KO
WB
HU
WB
HU
LepR/DAPI
d
WT
KO
LepR positivity
（percentage of DAPI）
0.5
0.4
0.3
0.2
0.1
0.0
***
***
WB
HU

Supplementary Fig. S4 (a) Representative images of tibia sections from WB- and HU-rats stained with ALP and TRAP. (b) Quantification of ALP and TRAP staining. The data are presented as percentages of the positively stained area relative to the total bone area. n=6. (c) Representative images of tibia sections from WB- and HU-rats stained with LepR. (d) Quantification of $LepR^+$ cells in tibia sections from rats subjected to unloading for 7 days. n=6. The data are presented as percentages of positively stained cells to total cells (DAPI-stained nuclei) *P<0.05, **P<0.01, and ***P<0.001. Statistical significance was assessed by Student`s t-test of the results from 3 independent experiments. The data are presented as means ± SDs.

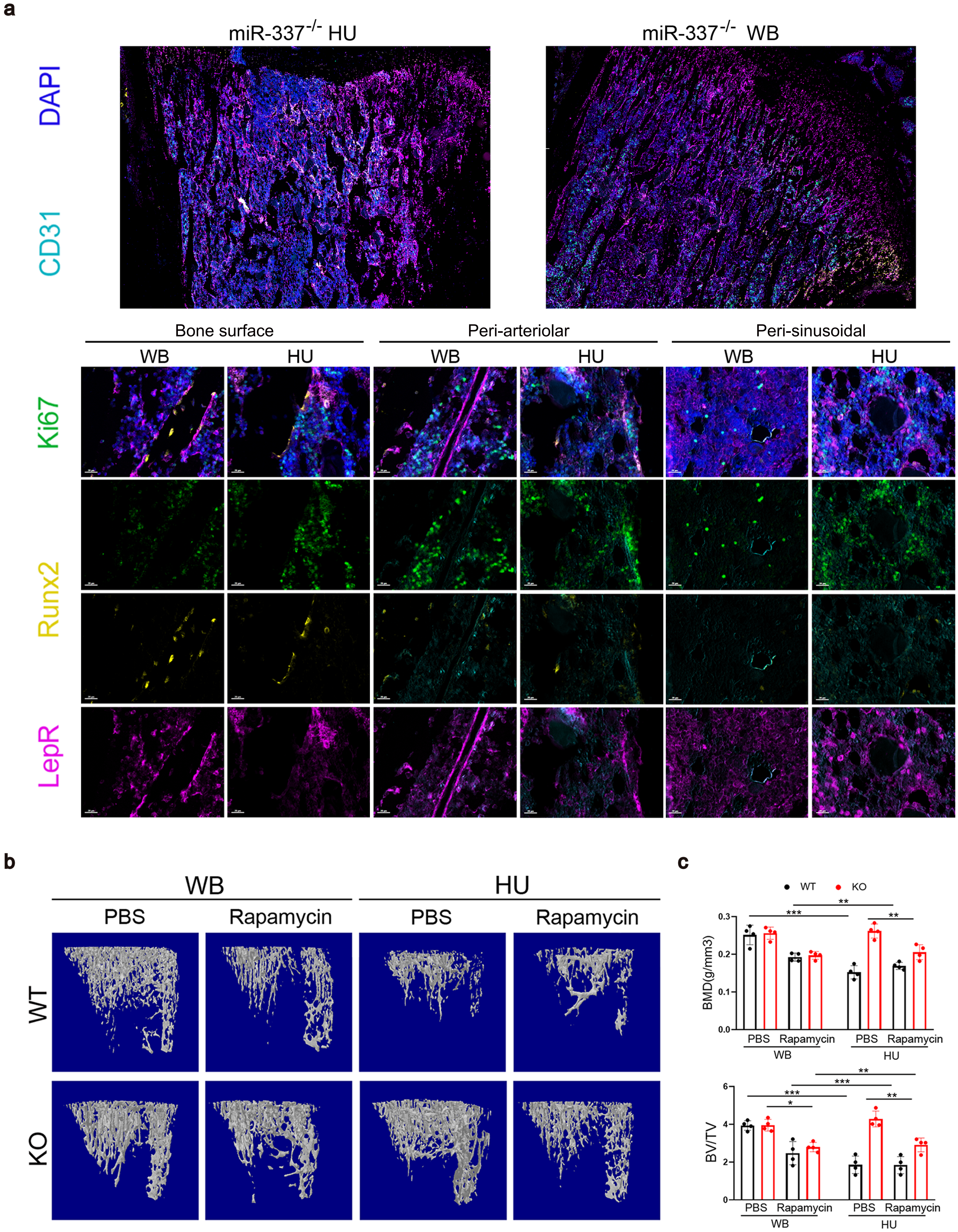
a
miR-337-/- HU
miR-337-/- WB
DAPI
CD31
Bone surface
Peri-arteriolar
Peri-sinusoidal
WB
HU
WB
HU
WB
HU
Ki67
Runx2
LepR
b
WB
HU
PBS
Rapamycin
PBS
Rapamycin
WT
KO
c
WT
KO
BMD(g/mm3)
0.3
0.2
0.1
0.0
***
**
**
PBS
Rapamycin
PBS
Rapamycin
WB
HU
BV/TV
6
4
2
0
**
***
***
*
**
PBS
Rapamycin
PBS
Rapamycin
WB
HU

Supplementary Fig. S5 (a)Representative images of mIHC staining of tibia sections from miR-337 KO rats after 7 days of tail suspension. Scale bars, (upper panel) 600 μm; (lower panel) 20 μm. (b) Representative micro-CT images of the bone parameters of WT or KO rats treated with rapamycin and subjected to tail-suspension for 28 days. (c) Quantification of three-dimensional microstructural parameters from micro-CT image of the tibia. The statistical significance of the results from the experiments shown in B-C was assessed by one-way ANOVA with Dunnett's post hoc test. **P*<0.05, ***P*<0.01, and ****P*<0.001. n=6 per group. The data are presented as means ± SEMs.

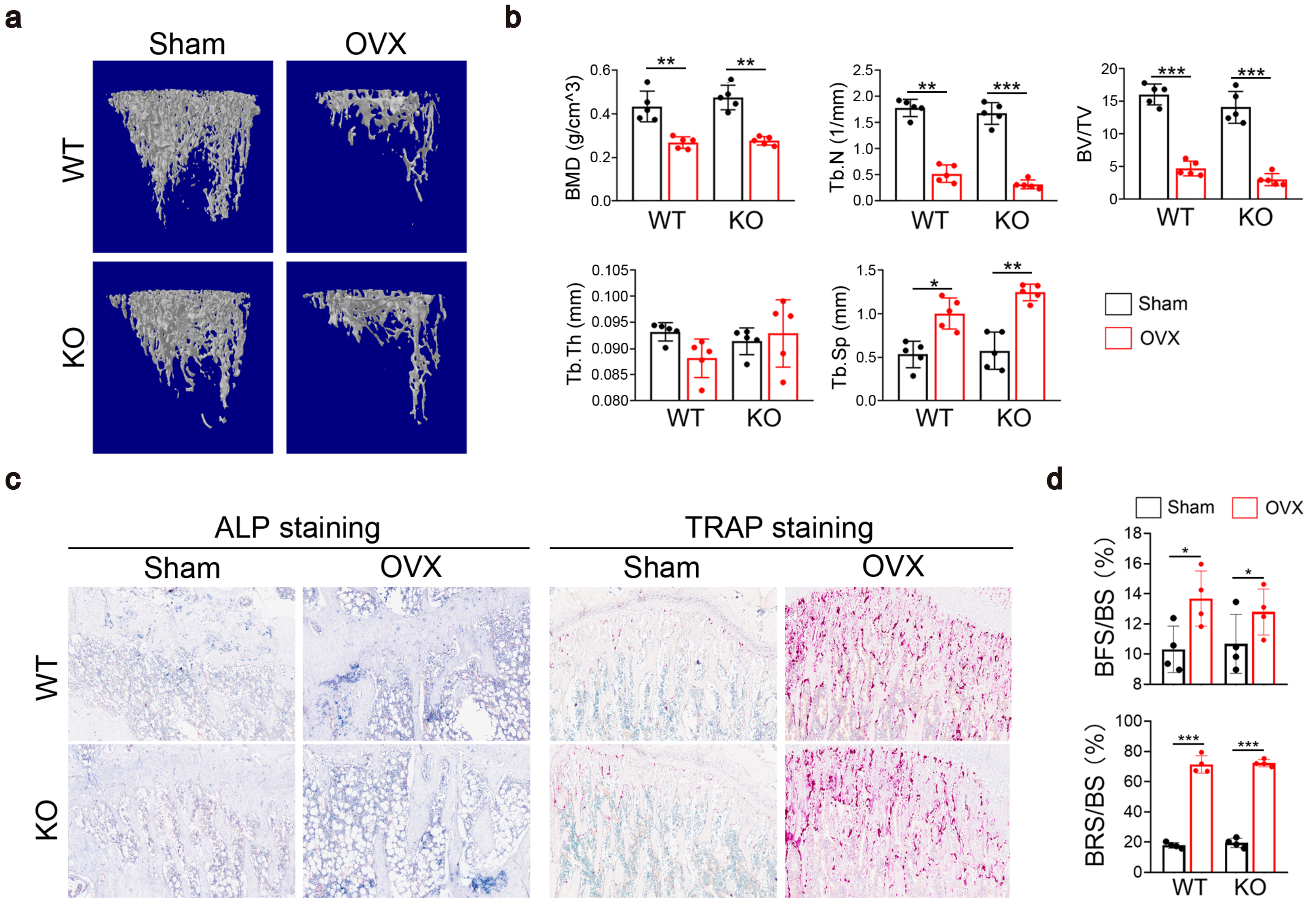
a
Sham
OVX
WT
KO
b
BMD (g/cm^3)
Tb.N (1/mm)
BV/TV
Tb.Th (mm)
Tb.Sp (mm)
WT
KO
Sham
OVX
c
ALP staining
TRAP staining
Sham
OVX
WT
KO
d
BFS/BS (%)
BRS/BS (%)
Sham
OVX
WT
KO

Supplementary Fig. S6 (a) Representative micro-CT images of tibiae from WT- and miR-337$^{-/-}$ female rats at 28 days after ovariectomized (OVX). (b) Quantification of three-dimensional microstructural parameters from micro-CT scans of the tibiae. (c) Representative images of ALP staining and TRAP staining of tibia sections from sham-/OVX- rats. (d) Quantification of ALP (BFS/BS) and TRAP (BRS/BS) staining. The data are presented as percentages of the positively stained area relative to the total bone area. n=4 per group. Statistical significance was assessed by two-tailed Student's t-test. *P<0.05, **P<0.01, and ***P<0.001 compared with the sham group. n=6 per group. The data are presented as the means ± SDs of 3 independent experiments.

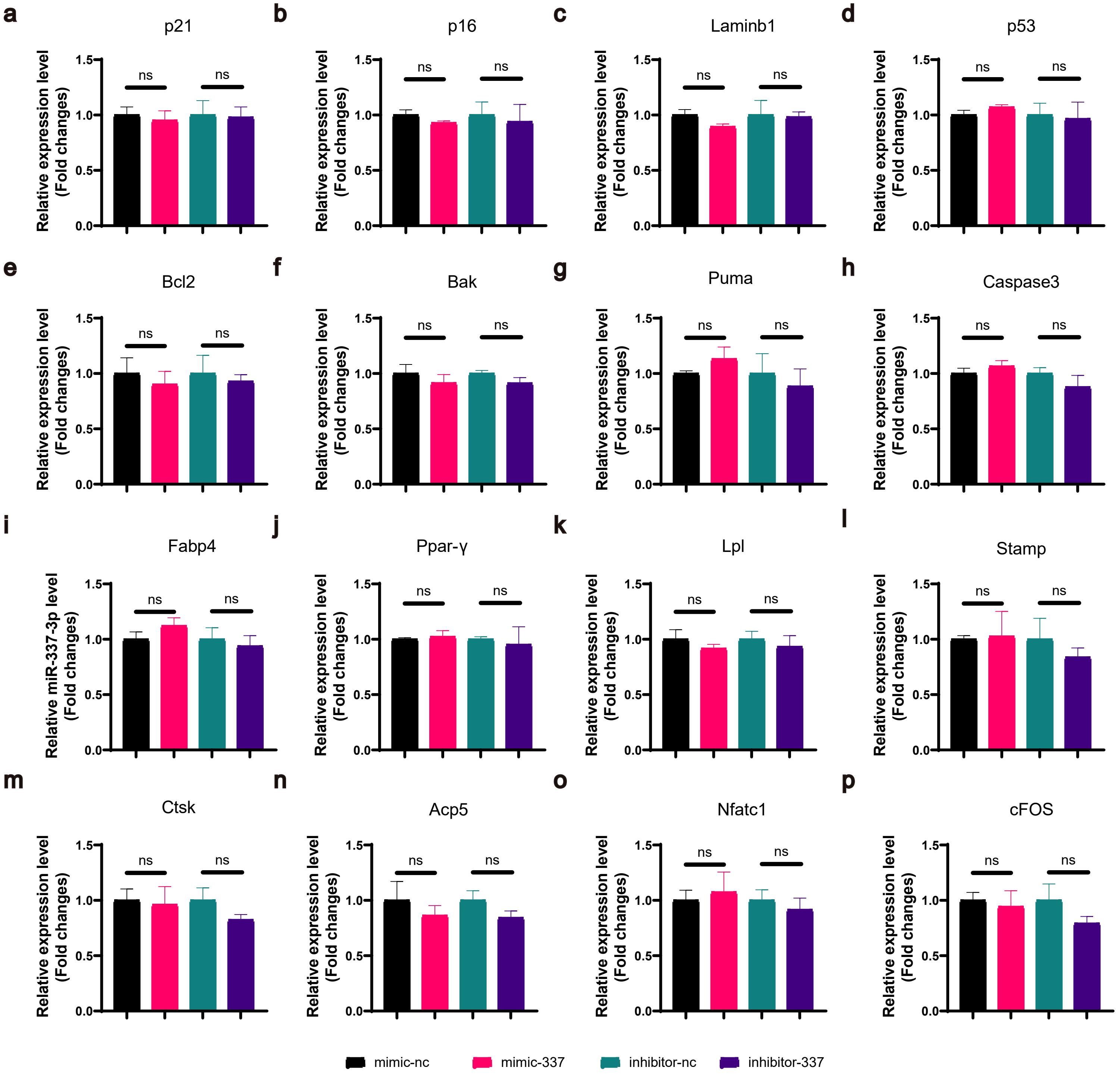
a p21
b p16
c Laminb1
d p53
e Bcl2
f Bak
g Puma
h Caspase3
i Fabp4
j Ppar-γ
k Lpl
l Stamp
m Ctsk
n Acp5
o Nfatc1
p cFOS
Relative expression level (Fold changes)
Relative miR-337-3p level (Fold changes)
ns
mimic-nc
mimic-337
inhibitor-nc
inhibitor-337

Supplementary Fig. S7 (a-h): Quantitative RT-PCR anlysis of the expression of senescence (a-d) and apoptosis (e-h) markers in rMSC at 72 h after transfection with the rat miR-337 mimics or inhibitor. (i-k): Quantitative RT-PCR was used to measure the expression of adipogenesis markers in rMSC. rMSC were transfected with rat miR-337 mimics or inhibitor as indicated for 24 h before the adipogenesis assay. (l-p): Quantitative RT-PCR analysis of the expression of osteoclastogenic markers in RAW264.7 cells. RAW264.7 cells were transfected with mouse miR-337 mimics or inhibitor as indicated for 24 h before osteoclastogenic assay. The data are normalized to the level of GAPDH. Statistical significance was assessed by Student`s t-test of the results from 3 independent experiments. The data are presented as the means ± SDs.
